# Plant and soil biodiversity sustain root mycorrhizal fungal richness under drought stress

**DOI:** 10.1093/ismejo/wraf102

**Published:** 2025-05-22

**Authors:** Markus Bittlingmaier, Nathalie Séjalon-Delmas, Kezia Goldmann, David Johnson, Raoul Huys, Grégoire T Freschet

**Affiliations:** Station d'Écologie Théorique et Expérimentale, CNRS, 2 route du CNRS, 09200 Moulis, France; LRSV, UPS, CNRS, Université de Toulouse, 24 chemin de Borde Rouge, 31320 Auzeville-Tolosane, France; Department of Soil Ecology, Helmholtz Centre for Environmental Research (UFZ), Theodor-Lieser-Straße 4, 06120 Halle/Saale, Germany; Lancaster Environment Centre, Lancaster University, Library Avenue, Lancaster LA1 4YQ, United Kingdom; Station d'Écologie Théorique et Expérimentale, CNRS, 2 route du CNRS, 09200 Moulis, France; Station d'Écologie Théorique et Expérimentale, CNRS, 2 route du CNRS, 09200 Moulis, France

**Keywords:** arbuscular mycorrhizal fungi, drought, root traits, plant–soil interactions, plant diversity, soil biodiversity, phylogenetic richness, temporal change

## Abstract

Mycorrhizal phenotypes arise from interactions among plants, soil biota, and environmental factors, but disentangling these drivers remains a key challenge in ecology. Understanding how these interactions shape mycorrhizal community assembly and stability is essential for predicting and managing these relationships in both natural and agricultural ecosystems. Here, we designed a fully factorial experiment examining how plant and soil biodiversity impact arbuscular mycorrhizal fungal communities under drought conditions. We further examined the role of plant ecological strategies in shaping these communities by including 16 herbaceous plant species along a gradient of plant-mycorrhizal reliance. Specifically, we investigated how plant traits and functional groups affected root-associated arbuscular mycorrhizal fungal richness and composition. Although drought decreased arbuscular mycorrhizal fungal phylogenetic species richness in roots, this effect was mitigated by higher soil and plant biodiversity. Plants with traits indicating high mycorrhizal reliance, such as legumes, displayed lower arbuscular mycorrhizal fungal richness but maintained higher constancy over time and across treatments. Overall, our findings indicate that ecosystems with limited plant and soil biodiversity partially lose their ability to support diverse arbuscular mycorrhizal root colonization under drought conditions. If repeated, such a loss could have severe implications for both immediate plant functioning and long-term soil health. The varied responses of arbuscular mycorrhizal fungal communities to drought in plants with differing ecological strategies suggest diverse fitness outcomes for plants and their symbionts, underscoring the need to integrate plant-symbiont dynamics into ecosystem management approaches to address global change.

## Graphical Abstract

**Graphical Abstract:**
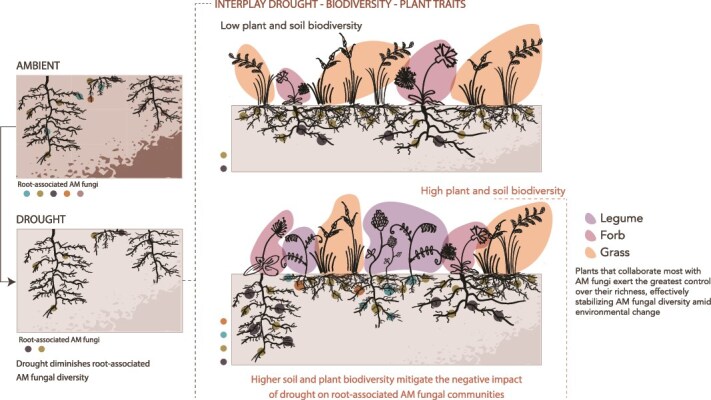

## Introduction

Understanding how plants adapt to environmental change is crucial for effective ecosystem management. Mycorrhizae—symbiotic associations between plant roots and fungi—play a central role in this adaptation [1, 2]. They facilitate nutrient exchange, sustain fungal networks, and enhance plant growth and stability [3–5]. However, the effects of mycorrhizal colonization on plant fitness can vary. They range from beneficial to detrimental, depending on specific interactions among plants, fungi, and environmental conditions [3, 6]. These interactions define the mycorrhizal phenotype, which represents the fitness outcomes for both partners [7]. Although research has identified key drivers of these phenotypes—such as nutrient availability and the evolutionary history of symbiotic partners—current understanding remains limited to specific plant-fungal pairings and particular environmental conditions [8]. Identifying the ecological processes that drive mycorrhizal community assembly across host plant species and conditions is key to understanding mycorrhizal phenotype formation, which is crucial for developing integrative perspectives on plant responses to environmental change.

Both mycorrhizal community assembly and mycorrhizal phenotypes are shaped by selection pressures from plants, fungi, and the environment [9, 10]. Arbuscular mycorrhizal (AM) fungi represent the most widespread mycorrhizal type [11] and are obligate symbionts with broad host ranges. This has led to the assumption that their assembly is largely govern by plant-driven constraints [12–14]. However, evidence of bidirectional selection in AM fungi shows that both plants and fungi display non-random association patterns and trait-based partner selection [10, 15, 16]. Environmental factors further influence this biotic filtering; e.g. nutrient availability and drought stress typically reduce plant investment in AM fungi [17]. Such deterministic filtering underscores the importance of symbiont diversity, as greater diversity among plants and AM fungi potentially increases the likelihood of beneficial plant-fungal pairings [10, 16].

Plant and soil biodiversity form a dynamic, interdependent system that affects the availability and effectiveness of plant-AM fungal relationships. For example, AM fungal diversity can alter plant diversity and plant-driven symbiont selection [10, 18, 19], whereas plant diversity co-drives fungal diversity by shaping niche spaces through varied resource provision and symbiont preferences [20–22]. Competition among AM fungi [23–25], and biotic interactions—such as those with root-feeding nematodes [9, 26]—can further disrupt preferential symbiont selection, potentially compromising mycorrhizal functionality. These interactions play a critical role in modulating how plants and ecosystems respond to environmental changes [27], emphasizing that studying these factors in isolation may obscure essential ecological processes and dynamics.

Plant traits, particularly those related to resource acquisition and allocation, are key to understanding how plants influence and are influenced by AM fungal communities, especially under environmental stressors like drought [28–30]. Plants that heavily rely on mycorrhizal collaboration for resource acquisition, typically have coarse, extensively colonized roots with few and short root hairs [31]. In contrast, species that collaborate less with AM fungi tend to have more branched root systems with longer and denser root hairs, which are better adapted for independent nutrient uptake [32]. Additionally, the degree of mycorrhizal collaboration itself may modulate plant responses to drought, as AM fungi can directly enhance the drought tolerance by regulating hormone profiles [33], and improving water and nutrient uptake [34, 35]. Thus, differences in root architecture reflect plant ecological strategies essential for understanding plant responses to drought and their broader impacts on ecosystem dynamics [36–38].

One major challenge in studying processes co-driven by plant and soil biodiversity is managing the difference in temporal and spatial scales. AM fungal propagules can remain dormant in soil for years, limiting bulk-soil biodiversity assessments to reflect long-term processes [39–41]. In contrast, root-associated fungal diversity captures dynamic, short-term interactions that better align with the scale relevant to mycorrhizal phenotypes. However, this approach introduces complexity by comparing biodiversity across different temporal scales, as plant developmental stages and environmental factors like seasonal variation continually shape AM fungal communities over the lifespan of a plant [42, 43]. Temporal dynamics thus become integral to understanding root-associated mycorrhizal diversity. Experimentally, a second challenge arises in manipulating soil biodiversity. Common methods, such as soil sterilization and reinoculation with limited taxa, poorly represent realistic diversity levels and disrupt natural microbial communities and essential interactions, including those between AM fungi and the hyphosphere microbiome [44–47]. Using non-sterilized soil or whole-soil inoculation offers greater ecological realism but complicates pinpointing specific organism groups or mechanisms.

Here, we aim to comprehensively assess the role of plant and soil biodiversity, including plant traits, in modulating drought effects on the richness and variability of AM fungal communities. We hypothesized that (i) root-associated AM fungal richness would increase with greater plant and soil biodiversity but decrease under drought conditions, with high plant and soil biodiversity buffering the negative effects of drought. We further hypothesized that (ii) plant functional groups and traits, as representative of plant ecological strategies (e.g. mycorrhizal reliance, carbon acquisition ability), would significantly and predictably influence the composition and richness of root-associated AM fungi. Finally, we hypothesized that (iii) plant traits, reflecting plant ecological strategies, would explain variations in the richness of root-associated AM fungi over time and in response to drought.

To test our hypotheses, we conducted two complementary mesocosm experiments to investigate how plant diversity, soil biodiversity, and drought interact to shape root-associated AM fungal communities, and to identify the biotic drivers most relevant to these interactions. First, we examined the combined effects of plant diversity, soil biodiversity, and drought stress on root-associated AM fungal communities, using eight perennial grassland plant species. This experiment employed a full-factorial design with two plant diversity levels (monocultures vs. six-species mixtures), two soil biodiversity conditions (based on an unsterilized but naturally low diversity sub-surface soil mixture inoculated with either sterile or live naturally high diversity surface soil), and two watering conditions (ambient and drought), with AM fungal diversity and composition assessed 15 months after sowing. To complement this experiment, we then broadened the scope to 16 herbaceous plant species, grown in monocultures and assessed at two distinct stages of vegetation maturity, to examine the role of plant traits in shaping the richness and temporal variability in root-associated AM fungal communities. Together, these experiments aimed to uncover the biotic drivers of root-associated AM fungal community assembly across varying environmental conditions.

## Material and methods

### Experimental setting

The two experiments comprised a total of 336 mesocosms, i.e. pots measuring 60 cm in height and 19 cm in diameter, wherein plants were grown in glasshouses for 15 months, from March 2021 until June 2022. Each pot contained six plant individuals from a species pool comprising 16 plant species (Table S1), including four legumes, six forbs, and six C3 grasses. Plant species were chosen for being representative of European temperate grasslands and spanning a wide variety of ecological strategies, functional types, and phylogenetic groups.

The soil substrate was a mix of 60% sand (<2 mm) and 40% non-sterilized grassland silt-clay soil (sieved at 10 mm) collected from a depth of 40–70 cm at a single site in southern France (November 2020; coordinates: 42.957°N, 1.087°E), which had low microbial diversity (confirmed via 16S and ITS metabarcoding). To establish two distinct soil biodiversity conditions, we inoculated this soil substrate with 2% (dry mass equivalent) of natural surface soil (0–20 cm) from the same site as the deep 40–70 cm horizon mentioned above. This whole-soil inoculum was sieved using a 2 mm mesh, with remaining root fragments removed. The soil substrate and whole-soil inoculum were thoroughly homogenized using a concrete mixer. For the low diversity condition, the whole-soil inoculum was sterilized beforehand (oven-dried at 200°C for 24 hours). The resulting mesocosm soil texture was 68% sand, 20% silt and 12% clay, with a pH of 8.2, low organic matter content (3.5 g kg^−1^), a cation-exchange capacity of 20.3 cmolc kg^−1^ and total N content of 0.25 g kg^−1^. The consistent use of non-sterilized soil substrate across all pots (98% dry mass equivalent), combined with sterilized versus non-sterilized inoculum, ensured uniform physical and chemical properties while maintaining a shared soil species pool between the two biodiversity treatments. By comparing pairwise operational taxonomic units (OTU) composition between the two soil biodiversity conditions, we found that high-diversity mycorrhizal communities shared, on average, 50.6% of their OTUs with low-diversity communities (calculated using Simpson’s similarity index), demonstrating the presence of a common base community between AM fungal communities in the low- and high-diversity treatments. Moreover, this biodiversity treatment provides a more ecologically realistic representation of contrasting soil biodiversity conditions compared to sterilization-based alternatives, better reflecting natural AM fungal taxa and soil microbial richness [44–46].

Chemical fertilizer was applied one, three, and five weeks after transplantation, as well as after seven months. This totalled 11 g N m^−2^, 11.5 g P m^−2^, 30 g K m^−2^, and 12 g S m^−2^ over the 15-month period. Preventive measures were taken against aphids, powdery mildew, and mites using organic farming treatments and predatory insects.

To represent natural conditions, glasshouse cell temperatures were allowed to fluctuate between 4°C and 35°C in parallel to outdoor conditions. Relative light homogeneity was ensured using diffractive transparent plastic sheeting. Watering followed two modalities: surface watering to mimic rainfall, and bottom watering from saucers to represent groundwater input. Water was added bi-weekly from the top in equal amount for all pots, following a seasonal pattern: 45 mm per week in spring and autumn, and 22 mm in summer and winter. Water in the saucers was regularly refilled but allowed to dry periodically during summer. Further details on the drought treatments are presented below.

Two (partly overlapping, i.e. some experimental units were shared) experiments were set up.

### Biodiversity–drought interaction experiment (240 mesocosms)

To assess the impact of soil biodiversity, plant diversity, drought, and their interactions on the root-associated AM fungal diversity, we established a full-factorial experiment. A selection of eight of the plant species (Table S1) was grown in monocultures and in six-species mixtures. Each pot was planted with six individuals, ensuring one individual per species in mixtures. To maintain consistency, plant individuals that failed to establish within three weeks were replaced to ensure six plants per pot. Monoculture mesocosms were replicated four times, while mixture mesocosms spanned all 28 possible plant species combinations without replication. This plant diversity treatment was crossed with the soil biodiversity treatment and a drought treatment simulating moderate drought conditions. This drought treatment was characterized by a severe reduction in precipitation combined with restricted plant access to the water table. It was applied for six consecutive weeks in early summer 2021 and again in 2022, with surface watering gradually reduced over two weeks before being completely halted, while bottom watering was restricted to the minimum necessary for plant survival across all eight species. During this period, water was added to saucers only when plants showed clear signs of severe drought stress, such as wilting. Overall, watering was reduced in two steps: first by 26%, then by 75%, resulting in a total reduction of 59% over six weeks compared to ambient conditions. The mesocosms were harvested in June 2022, 15 months after sowing. Aboveground biomass was collected at three intermediate time points to simulate meadow mowing.

### Plant ecological strategy experiment (128 mesocosms)

This experiment tested the relationships of plant species, functional groups, and traits with both AM fungal richness and its temporal variability. A total of 16 plant species were grown in monocultures under ambient watering and high soil biodiversity conditions with eight replicates per species. Half of these replicates were harvested after three months, and the other half after 15 months.

### Plant and soil analyses

A total of 17 plant traits were measured after a 3-month growth period on all 16 plant species grown in monocultures, as part of the plant ecological strategy experiment. Measurements were taken from three replicate mesocosms per species using standardized protocols [48–50]. These traits included five aboveground and 12 belowground traits (see Table S2 for details on all traits and their ecological significance). Of these, only five traits were included in the final statistical models after model selection and are briefly described here.

For aboveground plant traits, leaves and stems were harvested separately, weighed, oven-dried at 60°C for 48 h, and re-weighed. Leaf dry matter content (leaf dry matter content [LDMC], mg g^−1^) was measured as the ratio of leaf dry to fresh mass. For morphological root trait analysis, 10 undamaged root branches including the three most distal root orders were randomly sampled per species from the topsoil layer (0–20 cm). These fragments were placed on a transparent water tray, scanned at 800 dpi with an Epson Perfection V800 scanner, and weighed fresh and after oven-drying for 48 h at 60°C. Root images were analysed using RhizoVision Explorer (v2.0.3) [51] in order to estimate specific root length (SRL, m g^−1^), root tissue density (RTD, mg mm^−3^), and mean root diameter (MRD, mm). To measure root hair length (RHL, mm) pictures were taken under a stereomicroscope at 20× magnification and analysed using ImageJ. Root hair length was determined as the mean perpendicular distance from the root surface to the tip of the root hairs, based on 30 images as measurement replicates, excluding unusually long hairs. Dry root samples were ground and analysed for root nitrogen concentration (RNC, mg g^−1^) using an elemental analyser (CHN model EA1108; Carlo Erba Instruments, Milan, Italy). SRL, MRD, RTD, and RNC were summarized into the root economic space (Fig. S1) which consists of the root collaboration gradient (RCG1) and the root conservation gradient (RCG2; see *Statistical Analysis*). Finally, the shoot-to-root ratio (SR-ratio) was calculated as the aboveground plant dry mass divided by the total plant dry mass.

Samples for genetic analyses were taken after three and 15 months in the plant ecological strategy experiment, and after 15 months in the biodiversity–drought interaction experiment. Roots from the topsoil cylinders (0–20 cm), still attached to the plant base, were sorted by species and washed. Approximately 10 root branches, including the three most distal root orders, were randomly sampled per plant individual. From these, the first two root orders were further subsampled, cut into 1 cm pieces, and stored in 70% alcohol until DNA extraction.

### DNA extraction and sequencing

Root samples were flash-frozen in liquid nitrogen, ground, and sub-sampled to a dry weight equivalent of 200 mg. DNA extraction was performed separately for each plant species per mesocosm using the Qiagen’s DNeasy 96 Plant Kit, following the manufacturer’s protocol. DNA quality and yield were controlled using spectrophotometry. If the DNA content was low (<5 ng μL^−1^), the extraction was repeated. For plant mixtures, DNA extracts were pooled at the mesocosm level to ensure equal DNA content across plant species. To prevent the overrepresentation of mixtures relative to monocultures, DNA amounts and concentrations were standardized across both groups.

To characterize AM fungi, we employed 18S rRNA gene amplicon sequencing, with DNA samples processed by Argaly (Sainte-Hélène-du-Lac, France; https://www.argaly.com/). Specifically, Glom01 primers (forward sequence: ACTATCCCTATTAATCATTAC; reverse sequence: CTCGTAGTTGAATTTCG) were utilized to target a 216 bp fragment of the 18S rRNA gene region, with each amplification repeated four times. These primers were specifically designed to target AM fungi and underwent extensive quality control (Fig. S2) to ensure performance comparable to commonly used AM fungal primers like NS31/AML2 (Table S3).

The use of short-read sequencing enables the analysis of large sample sizes (1,276 purified and tagged PCR products), while balancing quality and quantity, though it may weaken the connection to AM fungal taxonomy and ecology compared to long-read sequencing [52]. To reduce potential biases from short reads, we employed specific bioinformatics tools like evolutionary placement algorithms (see Bioinformatics). Quality control measures, including negative and positive PCR controls as well as bioinformatic controls (to detect tag-jumping) were applied [53]. Purified amplicons were pooled and verified by capillary electrophoresis. Final amplicon mixtures were sent to Fasteris (Geneva, Switzerland; https://www.fasteris.com/dna/) for library preparation and amplification using the MetaFast protocol [54], followed by sequencing on a MiSeq System (Illumina; one MiSeq paired-end 2x150 V2 run and one MiSeq paired-end 2x150 V3 run).

### Bioinformatics

Sequencing reads were demultiplexed using OBITools3 (v3.0.1b26) [55], and processed into amplicon sequence variants (ASVs) using the DADA2 [56] based pipeline dadasnake (v0.4) [57]. Non-fungal ASVs were eliminated based on MOTHUR (v.1.48.0) [58] classification using the SILVA 138.1 SSU database. The remaining ASVs were subsequently clustered to OTU using unsupervised Bayesian clustering (CROP v1.33; [59]), applying a 2% similarity threshold.

Taxonomic classification of 18S rRNA gene OTU was conducted based on phylogenetic classification using parsimony-based phylogeny-aware short read alignment (PaPaRa v2.5; [60]) and evolutionary placement algorithm (EPA-ng v0.3.8; [61]). The corresponding reference tree was inferred using 86 consensus sequences [62, 63]. The alignment was calculated using the “L-INS-I” algorithm of MAFFT (ver. 7.520) [64]. A maximum likelihood tree was then calculated using RAxML-ng (v1.2.1) [65]. Bootstrap resampling was set to 1000 and the GTRGAMMA sequence evolutionary model was chosen. To test the sensitivity of that approach, comparative assessment of virtual taxa was conducted using the MaarjAM database [66]. Additionally, an alternative phylogenetic tree using only experiment-specific AM fungal OTU was inferred. For that, OTU core sequences were aligned using MAFFT (G-INS-i) and a maximum likelihood tree was constructed using the same conditions as for the reference tree.

### Statistical analysis

Analyses were performed in R (v4.4.0) [67]. Before analyses, contaminants were removed using the metabaR package (v1.0.0) [68]. Read tables were rarefied, and phylogenetic species richness [69]—hereafter “phylogenetic richness” (PR)—was calculated using the PICANTE package (v1.8.2) [70]. This metric quantifies sample-wise AM fungal OTU richness while accounting for OTU relatedness (sum of pairwise distances).

Zero-inflated negative-binomial generalized linear models (package GLMMTMB, v1.1.8; [71]) were employed to quantify the effect of the three main factors (i.e. plant diversity, soil biodiversity, and drought) and their two-way interactions on AM fungal richness, as well as to assess the relevance of plant traits. When the zero-inflation component was not significant, the model was simplified to a negative-binomial generalized linear model. Treatments were analysed using effect contrasts, with Type-II analysis of deviance if interactions were insignificant, or Type-III if interactions were significant (package car; v3.1.2; [72]).

The AM fungal community composition was analysed using non-rarefied read tables, respecting the compositional nature of the data [73]. OTU present in <1% of the samples were removed, and zero-imputation was performed using Bayesian-multiplicative replacement (zCompositions package; v1.5.0–1; [74]) to reduce bias from sparse compositional data. This count-table was then subjected to centred log-ratio (CLR) transformation (Aitchison Distance) using the vegan package (v2.6–4) [75]. Main effects were tested using permutational multivariate analysis of variance (PERMANOVA) and analysis of similarities (ANOSIM) to minimize bias from potential interactions. Both methods were adapted for three-way experimental designs and implemented in R, following an established methodology [76]. Effects of root traits on community composition were tested using redundancy analysis (RDA), whereas differences in dispersion were evaluated with a permutational test of multivariate homogeneity of group dispersions (PERMDISP).

To investigate the effect of plant traits on AM fungal diversity, plant traits were log-transformed, scaled, and centred. Root traits belonging to the root economic space (Fig. S1) were log-transformed before PCA, and principal components were scaled and centred after PCA.

Variability in AM fungal richness was defined as variability over time and treatments and was calculated species- and sample-wise using


(1) \begin{align*} \mathrm{AMF}\ \mathrm{Variability}\ \left(\%\right)=&\left|\left(\left({\mathrm{A}\mathrm{MF}}_{{\mathrm{Div}}_{\mathrm{A}}}-\mathrm{mean}\left(\mathrm{AM}{\mathrm{F}}_{Di{v}_B}\right)\right)\right.\right. \nonumber \\ &\left.\left.\times\, \mathrm{mean}{\left(\mathrm{AM}{\mathrm{F}}_{Di{v}_{AB}}\right)}^{-1}\right)\times 100\right| \end{align*}

This formula captures the difference between the observed AM fungal phylogenetic richness in condition A and the average richness in condition B, standardized by the overall average richness across both conditions. Conditions A and B represent the treatments being compared, which include temporal variability (A: 15 months, B: 3 months), soil-induced variability (A: low soil biodiversity, B: high soil biodiversity) and drought induced variability (A: drought, B: ambient). This data was analysed using generalized linear models assuming a quasipoisson distribution to examine the effects of plant traits on AM fungal variability, with group differences assessed using Tukey’s Honest Significant Difference test (Tukey’s HSD).

Model selection for statistical interactions and for plant traits was performed using forward selection based on the corrected Akaike Information Criterion. To address collinearity, the most representative traits were selected from among highly correlated candidates (see Table S4 for the correlation matrix of selected traits). Model assumptions were inspected by examining residuals using R packages performance (v0.10.9) [77] and DHARMa (v0.4.6) [78]. Asymptotic confidence intervals for pairwise comparisons were estimated using the emmeans package (v1.10.6) [79]. Adjusted *R*^2^ for zero inflated models were calculated using the performance package. To account for potential phylogenetic non-independence, we tested all plant species- and trait-related findings for phylogenetic signals using Pagel’s λ (phytools package, v2.4.4; [80]) and applied phylogenetic linear mixed modelling using the phyr package (v1.1.0) [81]. These models help control for shared evolutionary history among species, improving the robustness of trait-environment interaction estimates, even when the number of species is limited. The corresponding plant phylogenetic tree was constructed using the rtrees package (v1.0.3) [82]. Figures and data handling were done using tidyverse packages (v2.0.0) [83]. All assumptions and analyses underwent exhaustive sensitivity checks, detailed in the supplementary materials.

## Results

We extracted 879 root DNA samples and pooled them at the mesocosm level, resulting in 319 AM fungal 18S rRNA gene amplicon sequencing profiles. Among those, 24 samples (2.7%), mainly from mesocosms sampled after three months, were uncolonized. Sufficient sampling depth for all non-zero samples was confirmed using rarefaction curves. Following amplicon filtering, 114 OTUs remained (1707 amplicon sequence variants, belonging to 66 virtual taxa), spanning most currently described AM fungal families (Fig. S3). Overall, OTU richness per mesocosm ranged from one to 43, with similar species pools and extremes observed across both soil biodiversity levels.

### Effects of biodiversity and drought stress on root-associated AM fungal diversity

**Figure 1:**
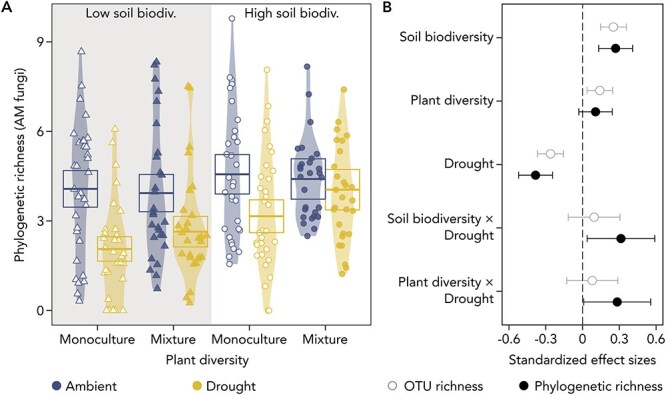
Responses of root-associated AM fungal diversity to the main treatments—soil biodiversity, plant diversity, drought—and their respective interactions (denoted by “×”). A: Violin plots depicting the distribution of phylogenetic richness across the three treatments. Each point represents a mesocosm (N = 240). The crossbars indicate the predicted mean, and the corresponding boxes depict the associated 95% asymptotic confidence intervals. B: Standardized effect sizes, representing relative changes, are plotted for phylogenetic richness and OTU richness of root-associated AM fungal communities. Estimates and 95% confidence intervals represent main effects of models using sum-to-zero contrasts.

Higher soil biodiversity significantly increased both the OTU richness (by 30.8%) and the phylogenetic richness (by 27.5%) of root-associated AM fungi (Fig. 1A and B, Table S5). Higher plant diversity led to a comparatively smaller increase (by 16.9%) in OTU richness. Drought caused a decline in both AM fungal OTU richness (by 26.5%) and phylogenetic richness (by 30.6%). Overall, the combined influence of all factors resulted in a 58% decrease in OTU richness of root-associated AM fungi and a corresponding 57.8% decrease in phylogenetic richness.

Significant interactions were found between drought and both soil and plant biodiversity in relation to AM fungal phylogenetic richness (Fig. 1A and B). AM fungal communities associated with plant mixtures in diverse soils experienced only a moderate drought-induced decrease in phylogenetic richness (by 12.2%). However, this decrease was significantly amplified under low soil biodiversity (by 41.9%), and a similar decrease (by 39.8%) in phylogenetic richness occurred under low plant diversity.

**Figure 2:**
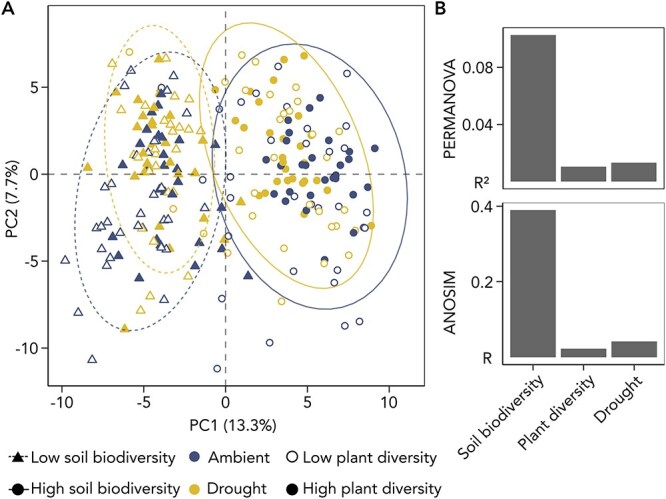
Effects of the plant diversity, soil biodiversity and drought treatments on root-associated AM fungal community composition after 15 months. A: Principal Component Analysis (PCA) using Aitchison distance to represent differences between AM fungal communities. Each point represents a mesocosm (N = 224). Ellipses depict the 95% data distribution for low and high soil biodiversity levels and watering condition. B: Impact of treatments on AM fungal community composition assessed through *R*^2^ values from PERMANOVA and R values from ANOSIM analyses. All plotted factors are significant (*P* value <0.05).

The soil biodiversity treatment was the strongest driver of AM fungal community composition, accounting for 10.3% of the observed variation in root-associated AM communities (Fig. 2A and B). In contrast, both plant diversity and drought contributed minimally to the observed variation in AM fungal community composition, each accounting for c. 1% of the variance.

### Effects of plant ecological strategy on root-associated AM fungal diversity

**Figure 3:**
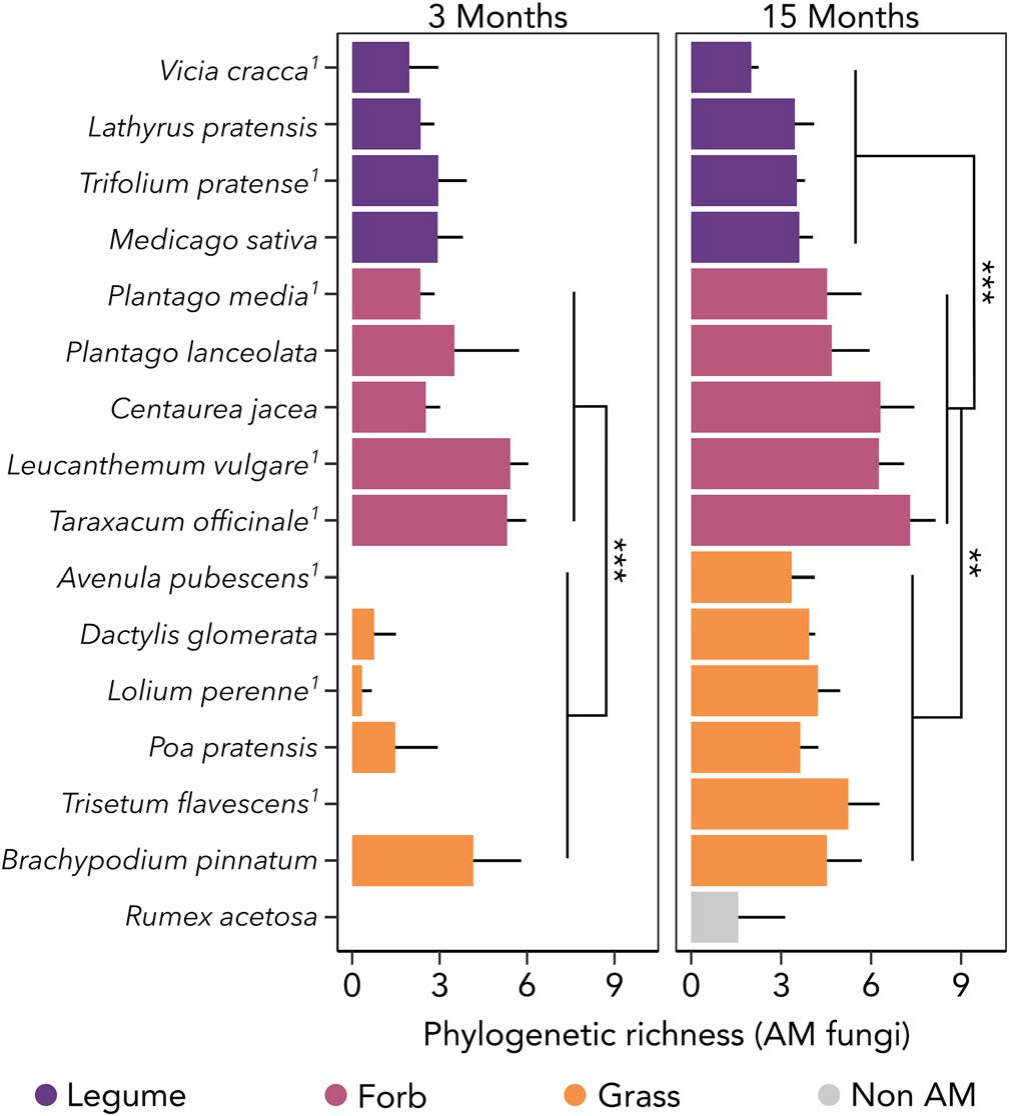
Influence of plant species and plant functional group on root-associated AM fungal phylogenetic richness at three and 15 months. Whiskers represent standard error. Significant differences between plant functional groups were assessed using Tukey’s HSD. Asterisks denote significance levels (^*^^*^^*^P value <0.001; ^*^^*^P value <0.01). Superscript “1” indicates plant species that were used in both experiments. N = 112.

**Figure 4:**
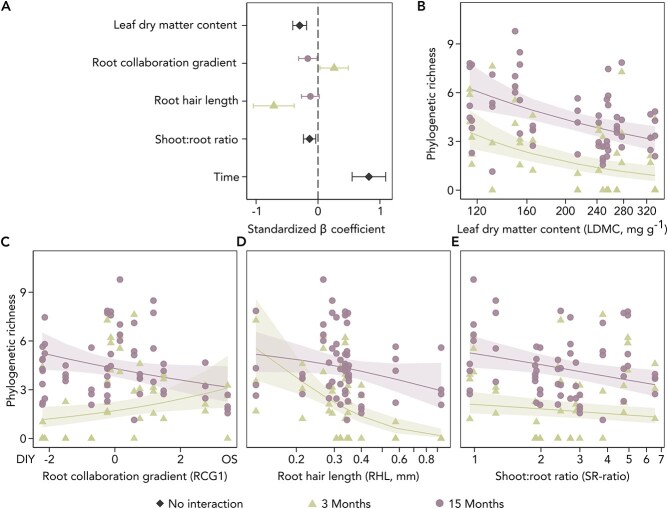
The impact of plant traits on root-associated AM fungal phylogenetic richness. Colours indicate measurement time points; non-AM plant species were removed. Each point represents a mesocosm (N = 105). A: Standardized β coefficients, using different colours to represent estimates at the two time points where interaction with time was significant. B-E: Plant traits plotted against AM fungal phylogenetic richness. Trend lines indicate a significant relationship between plant trait and observed AM fungal richness (*P* value <0.05). Leaf dry matter content (B), root hair length (D), and shoot-to-root ratio (E) are presented on a logarithmic scale. C: The root collaboration gradient, reflecting a gradient from a Do-It-Yourself (DIY) strategy, characterized by high specific root length and low mean root diameter, to an Outsourcing (OS) strategy, characterized by low specific root length and high mean root diameter.

Phylogenetic and OTU richness analyses yielded consistent results, with only the former presented here (see Tables S6–S7 for OTU richness). The forward-selected model for root-associated AM fungal phylogenetic richness (adjusted *R*^2^ = 0.74; Table S6) incorporated plant functional group (Fig. 3) and RHL. For plant traits only, the best model describing AM fungal phylogenetic richness (adjusted *R*^2^ = 0.74, Fig. 4A-E, Table S7) included RHL, LDMC, the RCG1 (representing SRL and MRD), and the SR-ratio. Specifically, longer RHL, higher LDMC and higher SR-ratio were associated with lower richness of root-associated AM fungi. The trend for RHL showed a significant decline in strength in the second measurement at 15 months. Higher scores on the root collaboration gradient were associated with greater AM fungal phylogenetic richness early on, but this relationship reversed over time. Specifically, at three months, plants with thicker fine roots exhibited significantly higher AM fungal richness, but at 15 months, this richness was lower compared to plants with thinner fine roots. As LDMC and RCG1 exhibited a phylogenetic signal, analyses were repeated using phylogenetic mixed-effects models, yielding fully consistent results (Tables S8–S9).

**Figure 5:**
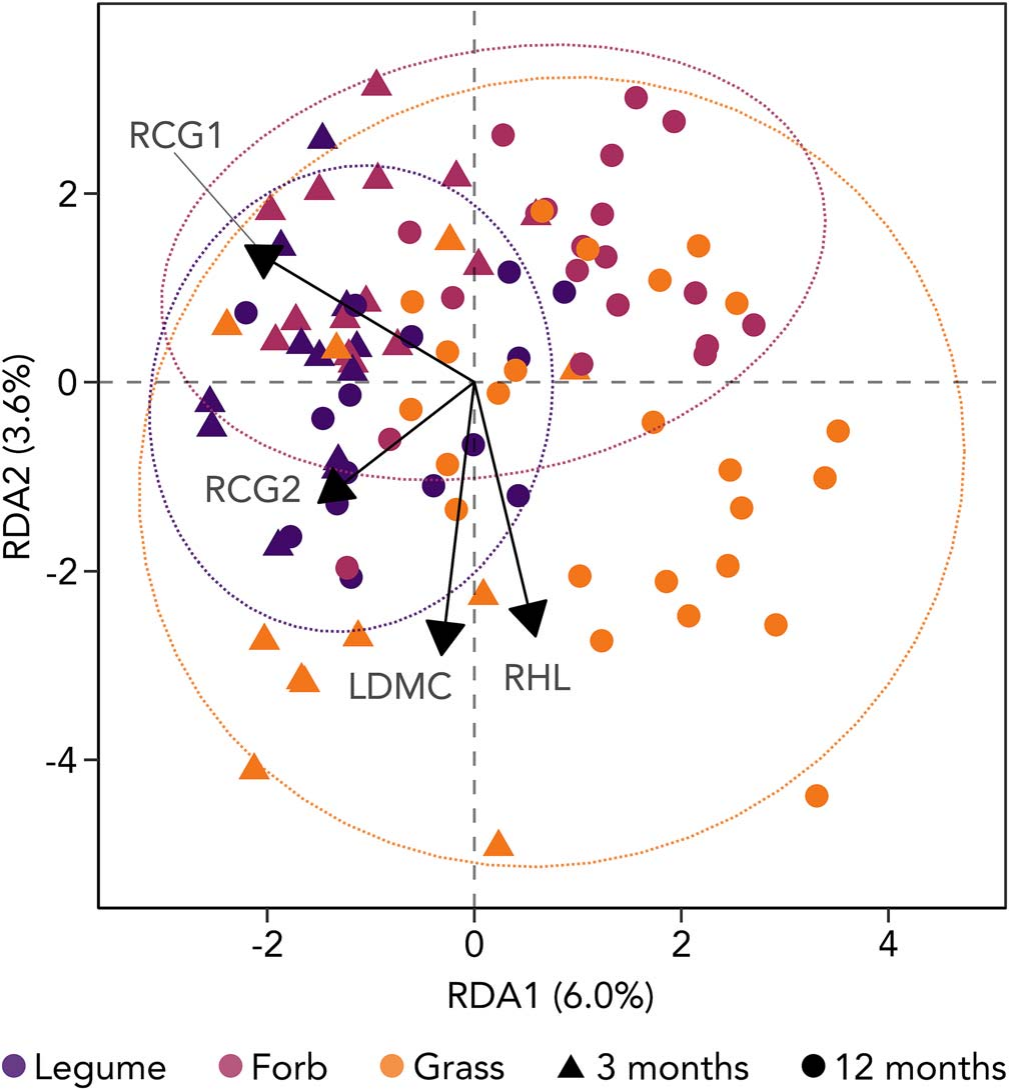
Redundancy analysis (RDA) of plant trait effects on root-associated AM fungal community composition in plant monocultures. Community distance is based on the Aitchison distance. This RDA includes the effect of time (depicted as shape), the root collaboration gradient (RCG1), the root conservation gradient (RCG2), root hair length (RHL), and leaf dry matter content (LDMC). Colours depict plant functional groups. Ellipses depict the 95% data distribution for plant functional groups. Non-AM plant species were removed. Each point represents a mesocosm, with 16 samples missing due to the absence of AM fungi in roots (N = 89).

Similarly to AM fungal phylogenetic richness, plant functional group and RHL most accurately explained root-associated AM fungal community composition, collectively accounting for 12% of the observed variation (adjusted *R*^2^ = 0.08). Effects of plant trait on root-associated AM fungal community composition (Fig. 5, Table S10) were primarily driven by the root collaboration gradient, along with the root conservation gradient (RCG2, representing RTD and RNC), RHL and LDMC, altogether explaining 12.7% (adjusted *R*^2^ = 0.08) of the observed variation. Additionally, dispersion in community composition was significantly higher in grasses than in forbs or legumes, a pattern consistent across both time points (PERMDISP, F = 15.50, *P* value <0.001; Table S11).

### Temporal and environmental variability of root-associated AM fungal diversity

Both root-associated AM fungal phylogenetic richness and community composition changed across the two sampling times. Phylogenetic richness increased by 85% in the one-year interval between samplings, with time being the most influential predictor in all previously discussed trait models (Fig. 4A). Similarly, time emerged as a main driver of AM fungal community composition across different RDA (with or without accounting for plant functional group), explaining 5% of the observed variability (Fig. 5).

**Figure 6:**
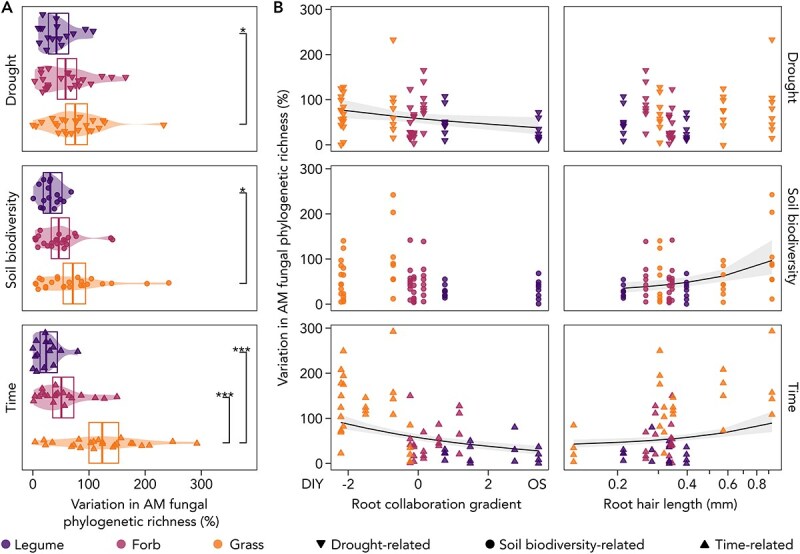
Environmental and temporal variability in root-associated AM fungal phylogenetic richness, represented as a percentage variation standardized by species-wise mean. Environmental variability (Soil biodiversity, Drought) encompasses 128 mesocosms across eight plant species, whereas temporal variability (Time) involves 105 mesocosms across 15 plant species. Non-AM plant species were removed. (A) Percentage variation in AM fungal phylogenetic richness across plant functional groups. Significant differences between plant functional groups (Tukey’s HSD) are indicated with asterisks (^*^^*^^*^P < 0.001; ^*^^*^P < 0.01; ^*^P < 0.05). B: Percentage variation in AM fungal phylogenetic richness modelled against the root collaboration gradient, ranging from Do-It-Yourself strategy (DIY; high specific root length, low mean root diameter) to Outsourcing strategy (OS; low specific root length, high mean root diameter), and root hair length (presented on a logarithmic scale). Significant linear relationships (*P* value <0.05) are represented as trend lines.

Temporal changes in AM fungal richness varied considerably across plant species and functional groups. While grasses and, to a lesser extent, forbs increased AM fungal richness between three and 15 months, richness in legumes remained relatively constant (Fig. 3). This pattern becomes particularly evident when examining temporal variability in AM fungal richness, which was best predicted by plant functional group (Fig. 6A, Table S7). Legumes and forbs exhibited greater constancy in AM fungal richness, with fluctuations of only 24% (legumes) and 51% (forbs) of their overall average over the one-year interval between samplings. In contrast, grasses showed significantly greater temporal variability in AM fungal richness, fluctuating by 124% of their overall average. Generally, species at the higher end of the root collaboration gradient and those with the shortest root hairs displayed the highest constancy in AM fungal richness over time (Fig. 6B, Table S9).

Environmentally driven variability in root-associated AM fungal richness was largely consistent with temporal variability (Fig. 6A and B). In response to change in soil biodiversity, AM fungal communities associated with legumes exhibited richness fluctuations of 31% of their overall average, significantly lower than that of grasses (71%). A similar trend was observed under drought conditions: the richness of root-associated AM fungal communities remained significantly more stable in legumes (fluctuating by only 42%) compared to grasses (fluctuating by 75%). These patterns were also reflected in plant traits: RHL was positively associated with the variability in AM fungal richness across soil biodiversity conditions, whereas increased stability under drought was linked to species’ positions along the root collaboration gradient.

## Discussion

The diversity of root-associated AM fungal communities is considered as an important driver of plant and ecosystem functioning [84, 85]. Yet, most research on factors shaping AM fungal diversity, particularly under climate stressors like drought, focuses on single variables such as plant diversity or soil properties, thereby overlooking their interactions. Here, we show that both higher soil and plant biodiversity mitigate the negative impact of drought on the phylogenetic richness of root-associated AM fungal communities. This suggests that ecosystems facing above- and below-ground biodiversity loss, combined with drought, may lose the ability to maintain diverse AM fungal colonization of plant roots. Plant functional group diversity is often seen as a key driver of AM fungal richness [22, 86]. Here, we demonstrate that beyond functional group, plant traits related to ecological strategy play a major role in shaping root-associated AM fungal diversity and its stability. Plant species that collaborate most with AM fungi and follow more resource conservative strategies [31] displayed the lowest richness of root-associated AM fungi. These species, especially legumes, also showed greater constancy over time and across treatments compared to less collaborative plants like grasses. Thus, plants that collaborate most with AM fungi contribute most effectively to stabilizing AM fungal richness under varying environmental conditions.

### Plant and soil biodiversity buffer drought effects on root-associated AM fungal phylogenetic richness

A positive effect of biodiversity, both above- and belowground, on the drought resistance of root-associated AM fungal diversity has been proposed but never demonstrated [40]. Here, focusing on short-term dynamics of root-associated AM fungi, we show that soil and plant biodiversity clearly buffer the drought-induced reduction in root-associated AM fungal phylogenetic richness (Fig. 1A and B). Under recurrent drought stress, such a reduction could have functional implications, as AM fungal phylogenetic richness is thought to affect plant and ecosystem functioning [45, 87], highlighting the importance of preserving or restoring plant- and soil biodiversity.

Improved drought resistance in diverse systems likely arises from both stochastic and deterministic mechanisms. In diverse soils, a high number of closely related OTUs may buffer against losses in phylogenetic richness, driven by greater phylogenetic redundancy and significantly clustered AM fungal communities (Fig. S4). Additionally, the ability of plants to filter symbionts and switch between partners in response to environmental and seasonal changes likely contributes to this resistance. Higher symbiont diversity may enhance mycorrhizal functioning by allowing plants to associate with AM fungi that are better adapted to prevailing environmental conditions [88].

In diverse plant communities, differences in ecological strategies and drought tolerance, along with resource use complementarity among plant species—for instance, through varying rooting depths [89, 90]—can improve water-use efficiency and help sustain carbon resource provision to AM fungi [91]. Our findings suggest that plant-mycorrhizal collaboration is a key factor in strengthening drought resistance in AM fungal communities within grasslands. The phylogenetic richness of AM fungal communities associated with plant species exhibiting high root collaboration gradient scores is the least affected by drought (Fig. 6). Due to the smaller number of plant species exposed to drought (eight species) this pattern cannot be entirely separated from differences between plant functional groups. Nevertheless, we emphasize that both factors—the root collaboration gradient and plant functional groups—suggest a selection effect, where the presence of highly mycorrhizal-reliant plant species [30] plays a crucial role in enhancing the drought resistance of AM fungal richness.

### Plant ecological strategies drive root-associated AM fungal richness

Plant traits provide valuable insights into the reciprocal relationship between plant ecological strategies and mycorrhizal communities. We found a negative relationship between LDMC, characterizing plants with slow metabolism and high defence, and AM fungal richness (Fig. 4B), supporting the idea that plants with strategies of resource conservation typically have lower photosynthetic capacity and allocate less carbon belowground [92, 93].

Three traits associated with plant-mycorrhizal reliance (RHL, SR-ratio, and the root collaboration gradient) showed a negative relationship with AM fungal richness, at least at the second sampling when AM fungal communities were fully established. First, plants on the more collaborative end of the root collaboration gradient supported lower AM fungal richness after 15 months (Fig. 4C), contradicting the hypothesis that greater collaboration increases AM fungal richness [94]. Second, the negative relationship between RHL and AM fungal richness (Fig. 4D) was strong at three months but had diminished in fully established communities. Longer root hairs are essential for plants with minimal mycorrhizal collaboration, enhancing direct acquisition of soil phosphorus and nitrogen [95]. Third, a higher SR-ratio was associated with lower AM fungal richness (Fig. 4E), potentially indicating greater carbon allocation to nutrient acquisition via mycorrhizal networks rather than being invested in the root system itself [96].

This inverse relationship between plant-mycorrhizal reliance and AM fungal richness aligns with findings on plant functional groups and mycorrhizal status. Recent research has demonstrated that the plant mycorrhizal growth response decreases from legumes to forbs to grasses, with legumes showing the highest mycorrhizal dependency [30]. Using a comparable selection of 16 plant species, we found this gradient reflected in root-associated AM fungal richness (Fig. 4A), with the most mycorrhizal-dependent group (legumes) associating with a more restricted range of AM fungal symbionts than less dependent groups like forbs and grasses, aligning with other studies [15, 97]. Research on plant mycorrhizal status further supports this trend, showing that obligately mycorrhizal species host less diverse AM fungal communities than facultatively mycorrhizal species [22].

Time emerged as an important factor, with results varying by sampling point. During plant establishment, plants with lower initial investment in AM fungi likely delayed the re-establishment of AM fungal communities in the disturbed soil of our experiment. This delay may have reduced mycorrhizal efficiency in supporting plant growth, further slowing AM fungal recruitment. This could explain the higher temporal variability in AM fungal plants with traits indicating lower mycorrhizal reliance, such as grasses (Fig. 6A and B). However, by the second growing seasons, AM fungal communities appeared fully re-established, even for the least collaborative plant species. This highlights the importance of considering interactions between environmental and biotic factors when assessing AM fungal diversity.

### Increased plant mycorrhizal reliance promotes constancy in root-associated AM fungal richness

Plants that heavily rely on AM fungi tend to form broad, generalist partnerships with their symbionts, resulting in greater dissimilarities between AM fungal communities across plants (beta diversity) but reduced AM fungal richness within plants (alpha diversity) [15, 22]. Although our experimental design, which focuses on AM fungal richness, limits our ability to address continuous turnover in AM fungal communities and fully capture beta diversity dynamics [98], our findings nevertheless support a pattern of reduced alpha diversity. We also observe that mycorrhizal-reliant plants exhibit increased constancy in AM fungal richness and seemingly less variation in community composition. Legumes, for instance, maintain more stable AM fungal richness across environmental conditions (i.e. soil biodiversity and drought treatments; Fig. 6A) and exhibit less dispersion in community composition compared to grasses, which show greater variability (Fig. 5). Directly linked to this pattern, plants with lower scores on the root collaboration gradient and longer root hairs exhibit higher variability in AM fungal richness (Fig. 6B). Together, these observations suggest that plants with traits indicating greater mycorrhizal reliance exert increased control over AM fungal communities, potentially through selective filtering and active recruitment [3, 99, 100]. Overall, such differences in the variability of AM fungal richness indicate that the species-specific costs and benefits defining the mycorrhizal phenotype—shaped by evolutionary and physiological factors—are tied to plant ecological strategies.

Beyond biotic drivers, environmental factors like water and nutrient availability influence AM fungal diversity [9], affecting AM fungal communities largely indirectly, as their effects are mediated by the host plant response and associated alteration in carbon provision [101]. Consistent with this idea, we show that plant functional group and ecological strategy are key predictors of AM fungal community response to environmental change (Fig. 6A and B). With only eight species included in the biodiversity–drought interaction conditions, it is challenging to distinguish the effects of plant functional group, phylogeny, and plant traits. Nevertheless, the overall pattern suggests that plant species less reliant on AM fungi reduce mycorrhizal diversity more strongly when environmental conditions become challenging. This difference may result from mycorrhizal-reliant plants continuing to invest heavily in fungal partners, even under unfavourable conditions.

## Conclusions and perspectives

Our study revealed substantial and predictable variation in the drought resistance of root-associated AM fungal communities, linked to plant ecological strategies and plant–soil biodiversity. Moreover, we found that plant species heavily reliant on AM fungi, such as legumes, exert the strongest control on root-associated AM fungal diversity. However, the role of AM fungal richness and composition in driving mycorrhizal phenotypes—and how these factors impact ecosystem functioning—remains to be explored. Overall, considering the decline in AM fungal diversity observed in highly disturbed agricultural soils worldwide, it is critical to further identify how such declines will differentially affect the fitness of plant with contrasting mycorrhizal strategies. Another challenge will be to understand if certain mycorrhizal strategies promote ecosystem functioning and stability, or whether stability arises from co-occurrence of contrasting mycorrhizal strategies.

## Supplementary Material

MS1_SI_final_wraf102

## Data Availability

The data supporting the findings of this study, along with the code used for analysis and figure generation, are accessible through the following links: 18S rRNA gene sequencing data: NCBI Sequence Read Archive, BioProject accession number PRJNA1189116 (https://www.ncbi.nlm.nih.gov/bioproject/PRJNA1189116). Data and R script: https://doi.org/10.6084/m9.figshare.28569116

## References

[1] Trivedi P, Batista BD, Bazany KE. et al. Plant–microbiome interactions under a changing world: responses, consequences and perspectives. *New Phytol* 2022;234:1951–9. 10.1111/nph.18016 35118660

[2] Vandenkoornhuyse P, Quaiser A, Duhamel M. et al. The importance of the microbiome of the plant holobiont. *New Phytol* 2015;206:1196–206. 10.1111/nph.13312 25655016

[3] Bennett AE, Groten K. The costs and benefits of plant-arbuscular mycorrhizal fungal interactions. *Annu Rev Plant Biol* 2022;73:649–72. 10.1146/annurev-arplant-102820-124504 35216519

[4] Martin FM, van der Heijden MGA. The mycorrhizal symbiosis: research frontiers in genomics, ecology, and agricultural application. *New Phytol* 2024;242:1486–506. 10.1111/nph.19541 38297461

[5] Smith SE, Read D. Mycorrhizal Symbiosis, 3rd edn. London: Academic Press, 2008.

[6] Johnson NC, Wilson GWT, Wilson JA. et al. Mycorrhizal phenotypes and the law of the minimum. *New Phytol* 2015;205:1473–84. 10.1111/nph.13172 25417818

[7] Johnson NC, Graham JH, Smith FA. Functioning of mycorrhizal associations along the mutualism-parasitism continuum. *New Phytol* 1997;135:575–85. 10.1046/j.1469-8137.1997.00729.x

[8] Lanfranco L, Fiorilli V, Gutjahr C. Partner communication and role of nutrients in the arbuscular mycorrhizal symbiosis. *New Phytol* 2018;220:1031–46. 10.1111/nph.15230 29806959

[9] Vályi K, Rillig MC, Hempel S. Community assembly and coexistence in communities of arbuscular mycorrhizal fungi. *ISME J* 2016;10:2341–51. 10.1038/ismej.2016.46 27093046 PMC5030697

[10] Werner GDA, Kiers ET. Partner selection in the mycorrhizal mutualism. *New Phytol* 2015a;205:1437–42. 10.1111/nph.13113 25421912

[11] Treseder KK, Cross A. Global distributions of arbuscular mycorrhizal fungi. *Ecosystems* 2006;9:305–16. 10.1007/s10021-005-0110-x

[12] Johnson D, Vandenkoornhuyse PJ, Leake JR. et al. Plant communities affect arbuscular mycorrhizal fungal diversity and community composition in grassland microcosms. *New Phytol* 2004; 161:503–15. 10.1046/j.1469-8137.2003.00938.x 33873500

[13] Krüger C, Kohout P, Janoušková M. et al. Plant communities rather than soil properties structure arbuscular mycorrhizal fungal communities along primary succession on a mine spoil. *Front Microbiol* 2017;8:719. 10.3389/fmicb.2017.00719 28473828 PMC5397529

[14] Hiiesalu I, Pärtel M, Davison J. et al. Species richness of arbuscular mycorrhizal fungi: associations with grassland plant richness and biomass. *New Phytol* 2014;203:233–44. 10.1111/nph.12765 24641509

[15] Sepp SK, Davison J, Jairus T. et al. Non-random association patterns in a plant–mycorrhizal fungal network reveal host–symbiont specificity. *Mol Ecol* 2019;28:365–78. 10.1111/mec.14924 30403423

[16] Chagnon PL, Bradley RL, Klironomos JN. Trait-based partner selection drives mycorrhizal network assembly. *Oikos* 2015;124:1609–16. 10.1111/oik.01987

[17] Wang R, Cavagnaro TR, Jiang Y. et al. Carbon allocation to the rhizosphere is affected by drought and nitrogen addition. *J Ecol* 2021;109:3699–709. 10.1111/1365-2745.13746

[18] Hart MM, Forsythe J, Oshowski B. et al. Hiding in a crowd—does diversity facilitate persistence of a low-quality fungal partner in the mycorrhizal symbiosis? *Symbiosis* 2013;59:47–56. 10.1007/s13199-012-0197-8

[19] Wagg C, Jansa J, Stadler M. et al. Mycorrhizal fungal identity and diversity relaxes plant–plant competition. *Ecology* 2011;92:1303–13. 10.1890/10-1915.1 21797158

[20] Martínez-García LB, Richardson SJ, Tylianakis JM. et al. Host identity is a dominant driver of mycorrhizal fungal community composition during ecosystem development. *New Phytol* 2015;205:1565–76. 10.1111/nph.13226 25640965

[21] Guzman A, Montes M, Hutchins L. et al. Crop diversity enriches arbuscular mycorrhizal fungal communities in an intensive agricultural landscape. *New Phytol* 2021;231:447–59. 10.1111/nph.17306 33638170 PMC9292320

[22] Davison J, García de León D, Zobel M. et al. Plant functional groups associate with distinct arbuscular mycorrhizal fungal communities. *New Phytol* 2020;226:1117–28. 10.1111/nph.16423 31943225

[23] Engelmoer DJP, Behm JE, Kiers ET. Intense competition between arbuscular mycorrhizal mutualists in an in vitro root microbiome negatively affects total fungal abundance. *Mol Ecol* 2014;23:1584–93. 10.1111/mec.12451 24050702

[24] Thonar C, Frossard E, Šmilauer P. et al. Competition and facilitation in synthetic communities of arbuscular mycorrhizal fungi. *Mol Ecol* 2014;23:733–46. 10.1111/mec.12625 24330316

[25] Werner GDA, Kiers ET. Order of arrival structures arbuscular mycorrhizal colonization of plants. *New Phytol* 2015b;205:1515–25. 10.1111/nph.13092 25298030

[26] Bell CA, Magkourilou E, Ault JR. et al. Phytophagy impacts the quality and quantity of plant carbon resources acquired by mutualistic arbuscular mycorrhizal fungi. *Nat Commun* 2024;15:801. 10.1038/s41467-024-45026-3 38280873 PMC10821877

[27] Rasmussen PU, Bennett AE, Tack AJM. The impact of elevated temperature and drought on the ecology and evolution of plant–soil microbe interactions. *J Ecol* 2020;108:337–52. 10.1111/1365-2745.13292

[28] Sweeney CJ, de Vries FT, van Dongen BE. et al. Root traits explain rhizosphere fungal community composition among temperate grassland plant species. *New Phytol* 2021;229:1492–507. 10.1111/nph.16976 33006139

[29] Brundrett MC . Coevolution of roots and mycorrhizas of land plants. *New Phytol* 2002;154:275–304. 10.1046/j.1469-8137.2002.00397.x 33873429

[30] Romero F, Argüello A, de Bruin S. et al. The plant–mycorrhizal fungi collaboration gradient depends on plant functional group. *Funct Ecol* 2023;37:2386–98. 10.1111/1365-2435.14395

[31] Bergmann J, Weigelt A, van der Plas F. et al. The fungal collaboration gradient dominates the root economics space in plants. *Sci Adv* 2020;6:eaba3756. 10.1126/sciadv.aba3756 32937432 PMC7458448

[32] Weigelt A, Mommer L, Andraczek K. et al. An integrated framework of plant form and function: the belowground perspective. *New Phytol* 2021;232:42–59. 10.1111/nph.17590 34197626

[33] Ruiz-Lozano JM, Aroca R, Zamarreño ÁM. et al. Arbuscular mycorrhizal symbiosis induces strigolactone biosynthesis under drought and improves drought tolerance in lettuce and tomato. *Plant Cell Environ* 2016;39:441–52. 10.1111/pce.12631 26305264

[34] Bowles TM, Jackson LE, Cavagnaro TR. Mycorrhizal fungi enhance plant nutrient acquisition and modulate nitrogen loss with variable water regimes. *Glob Chang Biol* 2018;24:e171–82. 10.1111/gcb.13884 28862782

[35] Kakouridis A, Hagen JA, Kan MP. et al. Routes to roots: direct evidence of water transport by arbuscular mycorrhizal fungi to host plants. *New Phytol* 2022;236:210–21. 10.1111/nph.18281 35633108 PMC9543596

[36] Müller LM, Bahn M. Drought legacies and ecosystem responses to subsequent drought. *Glob Chang Biol* 2022;28:5086–103. 10.1111/gcb.16270 35607942 PMC9542112

[37] Pérez-Ramos IM, Álvarez-Méndez A, Wald K. et al. Direct and indirect effects of global change on mycorrhizal associations of savanna plant communities. *Oikos* 2021;130:1370–84. 10.1111/oik.08451

[38] Eziz A, Yan Z, Tian D. et al. Drought effect on plant biomass allocation: a meta-analysis. *Ecol Evol* 2017;7:11002–10. 10.1002/ece3.3630 29299276 PMC5743700

[39] Cao Y, Li N, Lin J. et al. Root system–rhizosphere soil–bulk soil interactions in different Chinese fir clones based on fungal community diversity change. *Front Ecol Evol* 2022;10:1028686. 10.3389/fevo.2022.1028686

[40] Albracht C, Eisenhauer N, Vogel A. et al. Effects of recurrent summer droughts on arbuscular mycorrhizal and total fungal communities in experimental grasslands differing in plant diversity and community composition. *Front Soil Sci* 2023;3:1129845. 10.3389/fsoil.2023.1129845

[41] Nguyen NH, Hynson NA, Bruns TD. Stayin' alive: survival of mycorrhizal fungal propagules from 6-yr-old forest soil. *Fungal Ecol* 2012;5:741–6. 10.1016/j.funeco.2012.05.006

[42] Maciá-Vicente JG, Francioli D, Weigelt A. et al. The structure of root-associated fungal communities is related to the long-term effects of plant diversity on productivity. *Mol Ecol* 2023;32:3763–77. 10.1111/mec.16956 37081579

[43] Dumbrell AJ, Ashton PD, Aziz N. et al. Distinct seasonal assemblages of arbuscular mycorrhizal fungi revealed by massively parallel pyrosequencing. *New Phytol* 2011;190:794–804. 10.1111/j.1469-8137.2010.03636.x 21294738

[44] Kardol P, Throop HL, Adkins J. et al. A hierarchical framework for studying the role of biodiversity in soil food web processes and ecosystem services. *Soil Biol Biochem* 2016;102:33–6. 10.1016/j.soilbio.2016.05.002

[45] Hazard C, Johnson D. Does genotypic and species diversity of mycorrhizal plants and fungi affect ecosystem function? *New Phytol* 2018;220:1122–8. 10.1111/nph.15010 29393517

[46] Changey F, Meglouli H, Fontaine J. et al. Initial microbial status modulates mycorrhizal inoculation effect on rhizosphere microbial communities. *Mycorrhiza* 2019;29:475–87. 10.1007/s00572-019-00914-1 31506745

[47] Wang L, George TS, Feng G. Concepts and consequences of the hyphosphere core microbiome for arbuscular mycorrhizal fungal fitness and function. *New Phytol* 2024;242:1529–33. 10.1111/nph.19396 38044555

[48] Freschet GT, Pagès L, Iversen CM. et al. A starting guide to root ecology: strengthening ecological concepts and standardising root classification, sampling, processing and trait measurements. *New Phytol* 2021a;232:973–1122. 10.1111/nph.17572 34608637 PMC8518129

[49] Pérez-Harguindeguy N, Díaz S, Garnier E. et al. Corrigendum: new handbook for standardised measurement of plant functional traits worldwide. *Aust J Bot* 2016;64:715–6. 10.1071/BT12225_CO

[50] Brundrett M, Bougher N, Dell B. et al. Working with Mycorrhizas in Forestry and Agriculture. Canberra: Australian Centre for International Agricultural Research, 1996.

[51] Seethepalli A, Dhakal K, Griffiths M. et al. RhizoVision explorer: open-source software for root image analysis and measurement standardization. *AoB Plants* 2021;13:plab056. 10.1093/aobpla/plab056 34804466 PMC8598384

[52] Tedersoo L, Mikryukov V, Zizka A. et al. Global patterns in endemicity and vulnerability of soil fungi. *Glob Chang Biol* 2022;28:6696–710. 10.1111/gcb.16398 36056462 PMC9826061

[53] Schnell IB, Bohmann K, Gilbert MTP. Tag jumps illuminated—reducing sequence-to-sample misidentifications in metabarcoding studies. *Mol Ecol Resour* 2015;15:1289–303. 10.1111/1755-0998.12402 25740652

[54] Taberlet P, Bonin A, Zinger L. et al. Environmental DNA: For Biodiversity Research and Monitoring. Oxford: Oxford University Press; 2018. 10.1093/oso/9780198767220.001.0001

[55] Boyer F, Mercier C, Bonin A. et al. Obitools: a unix-inspired software package for DNA metabarcoding. *Mol Ecol Resour* 2016;16:176–82. 10.1111/1755-0998.12428 25959493

[56] Callahan BJ, McMurdie PJ, Rosen MJ. et al. DADA2: high-resolution sample inference from Illumina amplicon data. *Nat Methods* 2016;13:581–3. 10.1038/nmeth.3869 27214047 PMC4927377

[57] Weißbecker C, Schnabel B, Heintz-Buschart A. Dadasnake, a snakemake implementation of DADA2 to process amplicon sequencing data for microbial ecology. *Gigascience* 2020;9:giaa135. 10.1093/gigascience/giaa135 33252655 PMC7702218

[58] Schloss PD, Westcott SL, Ryabin T. et al. Introducing mothur: open-source, platform-independent, community-supported software for describing and comparing microbial communities. *Appl Environ Microbiol* 2009;75:7537–41. 10.1128/aem.01541-09 19801464 PMC2786419

[59] Hao X, Jiang R, Chen T. Clustering 16S rRNA for OTU prediction: a method of unsupervised Bayesian clustering. *Bioinformatics* 2011;27:611–8. 10.1093/bioinformatics/btq725 21233169 PMC3042185

[60] Berger SA, Stamatakis A. Aligning short reads to reference alignments and trees. *Bioinformatics* 2011;27:2068–75. 10.1093/bioinformatics/btr320 21636595

[61] Barbera P, Kozlov AM, Czech L. et al. EPA-ng: massively parallel evolutionary placement of genetic sequences. *Syst Biol* 2019;68:365–9. 10.1093/sysbio/syy054 30165689 PMC6368480

[62] Krüger M, Krüger C, Walker C. et al. Phylogenetic reference data for systematics and phylotaxonomy of arbuscular mycorrhizal fungi from phylum to species level. *New Phytol* 2012;193:970–84. 10.1111/j.1469-8137.2011.03962.x 22150759

[63] Stefani F, Bencherif K, Sabourin S. et al. Taxonomic assignment of arbuscular mycorrhizal fungi in an 18S metagenomic dataset: a case study with saltcedar (Tamarix aphylla). *Mycorrhiza* 2020;30:243–55. 10.1007/s00572-020-00946-y 32180012

[64] Katoh K, Misawa K, Kuma K. et al. MAFFT: a novel method for rapid multiple sequence alignment based on fast Fourier transform. *Nucleic Acids Res* 2002;30:3059–66. 10.1093/nar/gkf436 12136088 PMC135756

[65] Kozlov AM, Darriba D, Flouri T. et al. RAxML-NG: a fast, scalable and user-friendly tool for maximum likelihood phylogenetic inference. *Bioinformatics* 2019;35:4453–5. 10.1093/bioinformatics/btz305 31070718 PMC6821337

[66] Öpik M, Vanatoa A, Vanatoa E. et al. The online database MaarjAM reveals global and ecosystemic distribution patterns in arbuscular mycorrhizal fungi (Glomeromycota). *New Phytol* 2010; 188:223–41. 10.1111/j.1469-8137.2010.03334.x 20561207

[67] R Core Team. R: A Language and Environment for Statistical Computing. Vienna, Austria: R Foundation for Statistical Computing, 2021. https://www.R-project.org.

[68] Zinger L, Lionnet C, Benoiston AS. et al. metabaR: an R package for the evaluation and improvement of DNA metabarcoding data quality. *Methods Ecol Evol* 2021;12:586–92. 10.1111/2041-210X.13552

[69] Helmus MR, Bland TJ, Williams CK. et al. Phylogenetic measures of biodiversity. *Am Nat* 2007;169:E68–83. 10.1086/511334 17230400

[70] Kembel SW, Cowan PD, Helmus MR. et al. Picante: R tools for integrating phylogenies and ecology. *Bioinformatics* 2010;26:1463–4. 10.1093/bioinformatics/btq166 20395285

[71] Brooks ME, Kristensen K, van Benthem KJ. et al. glmmTMB balances speed and flexibility among packages for zero-inflated generalized linear mixed modeling. *R J* 2017;9:378–400. 10.32614/RJ-2017-066

[72] Fox J, Weisberg S. An R Companion to Applied Regression. Thousand Oaks: Sage Publications, 2018.

[73] Gloor GB, Macklaim JM, Pawlowsky-Glahn V. et al. Microbiome datasets are compositional: and this is not optional. *Front Microbiol* 2017;8:2224. 10.3389/fmicb.2017.02224 29187837 PMC5695134

[74] Palarea-Albaladejo J, Martín-Fernández JA. zCompositions—R package for multivariate imputation of left-censored data under a compositional approach. *Chemom Intell Lab Syst* 2015;143:85–96. 10.1016/j.chemolab.2015.02.019

[75] Oksanen J, Simpson GL, Blanchet FG. et al. *Vegan: community ecology package.* R package version 2.6-4. 2022;20.

[76] Somerfield PJ, Clarke KR, Gorley RN. Analysis of similarities (ANOSIM) for 3-way designs. *Austral Ecol* 2021;46:927–41. 10.1111/aec.13083

[77] Lüdecke D, Ben-Shachar MS, Patil I. et al. Performance: an R package for assessment, comparison and testing of statistical models. *J Open Source Softw* 2021;6:3139. 10.21105/joss.03139

[78] Hartig F, Karimi A, Evrard HC. DHARMa: *Residual diagnostics for hierarchical (multi-level/mixed) regression models.* R package version 0.4.6; 2022. 10.32614/CRAN.package.DHARMa, 10.3389/fnins.2022.818800, Interconnected sub-networks of the macaque monkey gustatory connectome, 16. PMC9978403 36874640

[79] Lenth R . Emmeans: estimated marginal means, aka least-squares means. *R package version* 2024;1.10.6. 10.32614/CRAN.package.emmeans

[80] Revell LJ . Phytools: an R package for phylogenetic comparative biology (and other things). *Methods Ecol Evol* 2012;3:217–23. 10.1111/j.2041-210X.2011.00169.x

[81] Li D, Dinnage R, Nell LA. et al. Phyr: an R package for phylogenetic species-distribution modelling in ecological communities. *Methods Ecol Evol* 2020;11:1455–63. 10.1111/2041-210X.13471

[82] Li D . rtrees: An R package to assemble phylogenetic trees from megatrees. *Ecography* 2023;7:e06643. 10.1111/ecog.06643

[83] Wickham H, Averick M, Bryan J. et al. Welcome to the tidyverse. *J Open Source Softw* 2019;4:1686. 10.21105/joss.01686

[84] Van der Heijden MGA, Klironomos JN, Ursic M. et al. Mycorrhizal fungal diversity determines plant biodiversity, ecosystem variability and productivity. *Nature* 1998;396:69–72. 10.1038/23932

[85] Powell JR, Rillig MC. Biodiversity of arbuscular mycorrhizal fungi and ecosystem function. *New Phytol* 2018;220:1059–75. 10.1111/nph.15119 29603232

[86] Cline LC, Hobbie SE, Madritch MD. et al. Resource availability underlies the plant-fungal diversity relationship in a grassland ecosystem. *Ecology* 2018;99:204–16. 10.1002/ecy.2075 29106700

[87] Yang H, Zhang Q, Koide RT. et al. Taxonomic resolution is a determinant of biodiversity effects in arbuscular mycorrhizal fungal communities. *J Ecol* 2017;105:219–28. 10.1111/1365-2745.12655

[88] Argüello A, O'Brien MJ, van der Heijden MGA. et al. Options of partners improve carbon for phosphorus trade in the arbuscular mycorrhizal mutualism. *Ecol Lett* 2016;19:648–56. 10.1111/ele.12601 27074533

[89] Oram NJ, Ravenek JM, Barry KE. et al. Below-ground complementarity effects in a grassland biodiversity experiment are related to deep-rooting species. *J Ecol* 2018;106:265–77. 10.1111/1365-2745.12877

[90] Van Peer L, Nijs I, Reheul D. et al. Species richness and susceptibility to heat and drought extremes in synthesized grassland ecosystems: compositional vs physiological effects. *Funct Ecol* 2004;18:769–78. 10.1111/j.0269-8463.2004.00901.x

[91] Balachowski JA, Volaire FA. Implications of plant functional traits and drought survival strategies for ecological restoration. *J Appl Ecol* 2018;55:631–40. 10.1111/1365-2664.12979

[92] Henneron L, Cros C, Picon-Cochard C. et al. Plant economic strategies of grassland species control soil carbon dynamics through rhizodeposition. *J Ecol* 2020;108:528–45. 10.1111/1365-2745.13276

[93] Han M, Chen Y, Sun L. et al. Linking rhizosphere soil microbial activity and plant resource acquisition strategy. *J Ecol* 2023;111:875–88. 10.1111/1365-2745.14067

[94] Hennecke J, Bassi L, Mommer L. et al. Responses of rhizosphere fungi to the root economics space in grassland monocultures of different age. *New Phytol* 2023;240:2035–49. 10.1111/nph.19261 37691273

[95] Freschet GT, Roumet C, Comas LH. et al. Root traits as drivers of plant and ecosystem functioning: current understanding, pitfalls and future research needs. *New Phytol* 2021b;232:1123–58. 10.1111/nph.17072 33159479

[96] Henkes GJ, Kandeler E, Marhan S. et al. Interactions of mycorrhiza and protists in the rhizosphere systemically alter microbial community composition, plant shoot-to-root ratio and within-root system nitrogen allocation. *Front Environ Sci* 2018;6:117. 10.3389/fenvs.2018.00117

[97] Ramana JV, Tylianakis JM, Ridgway HJ. et al. Root diameter, host specificity and arbuscular mycorrhizal fungal community composition among native and exotic plant species. *New Phytol* 2023;239:301–10. 10.1111/nph.18911 36967581

[98] Gao C, Montoya L, Xu L. et al. Strong succession in arbuscular mycorrhizal fungal communities. *ISME J* 2019;13:214–26. 10.1038/s41396-018-0264-0 30171254 PMC6298956

[99] Kiers ET, Duhamel M, Beesetty Y. et al. Reciprocal rewards stabilize cooperation in the mycorrhizal symbiosis. *Science* 2011;333:880–2. 10.1126/science.1208473 21836016

[100] Grman E . Plant species differ in their ability to reduce allocation to non-beneficial arbuscular mycorrhizal fungi. *Ecology* 2012;93:711–8. 10.1890/11-1358.1 22690621

[101] Fu W, Chen B, Rillig MC. et al. Community response of arbuscular mycorrhizal fungi to extreme drought in a cold-temperate grassland. *New Phytol* 2022;234:2003–17. 10.1111/nph.17692 34449895

