# Supplementary material for: Plant and soil biodiversity sustain root mycorrhizal fungal richness under drought stress
Source: ISME J. 2025 May 22;20(1):wraf102. doi: 10.1093/ismejo/wraf102 (PMC13019693; doi:10.1093/ismejo/wraf102)
Supplement: MS1_SI_final_wraf102 [file ms1_si_final_wraf102.pdf]

Supplementary Material

For publication in conjunction with the following:

Plant and soil biodiversity sustain root mycorrhizal fungal richness under drought stress

Markus Bittlingmaier[1], Nathalie Séjalon-Delmas[2], Kezia Goldmann[3], David Johnson[4], Raoul Huys[1], Grégoire T. Freschet[1]

[1]Station d'Écologie Théorique et Expérimentale, CNRS, Moulis, France
[2]LRSV, UPS, CNRS, Université de Toulouse, Toulouse, France
[3]Department of Soil Ecology, Helmholtz Centre for Environmental Research (UFZ), Halle/Saale, Germany
[4]Lancaster Environment Centre, Lancaster University, Lancaster, UK

Correspondence:
Markus Bittlingmaier, bittlingmaier@posteo.de

<u>Table of Contents:</u>

Figure S1: Biplot of the root economic space.

Figure S2: Conservation and specificity of the Glom01 primer set for AM fungi.

Figure S3: RAxML phylogenetic tree based on evolutionary placement of OTU core sequences on reference tree.

Figure S4: Nearest taxon index and net relatedness index for AM fungal communities.

Tables

Table S1: List of plant species used and the experiments in which they appeared.

Table S2: List of plant traits measured after three months and their ecological relevance.

Table S3: Specificity and taxonomic resolution of two primer pairs targeting AM fungi.

Table S4: Correlation among selected plant traits used to model AM fungal diversity and variability.

Table S5: Analysis of deviance tables for different diversity indices addressing biodiversity–drought interactions.

Table S6: Plant trait and functional group model results for different diversity indices.

Table S7: Plant trait model results for different diversity indices.

Table S8: Pagel's λ indicating the phylogenetic signal in variables across all 16 plant species.

Table S9: Phylogenetic linear mixed models testing trait–AM fungal diversity relationships, accounting for phylogenetic signal.

Table S10: RDA model selection addressing plant trait effects on AM fungal community composition.

Table S11: Pairwise differences in dispersion among the three plant functional groups at three months, 15 months, and across both time points, assessed using Tukey's test.

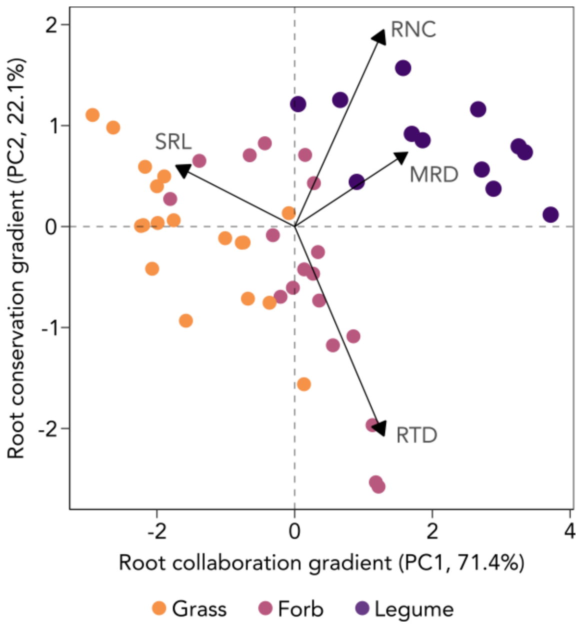

Fig. S1: Root economic space, calculated using principal component analysis of log-transformed root traits, namely root tissue density (RTD), mean root diameter (MRD), root nitrogen content (RNC), and specific root length (SRL). Each point represents an individual replicate (N=48), which were subsequently averaged to define mean species score on each axis (N=16). Colours indicate different plant functional groups.

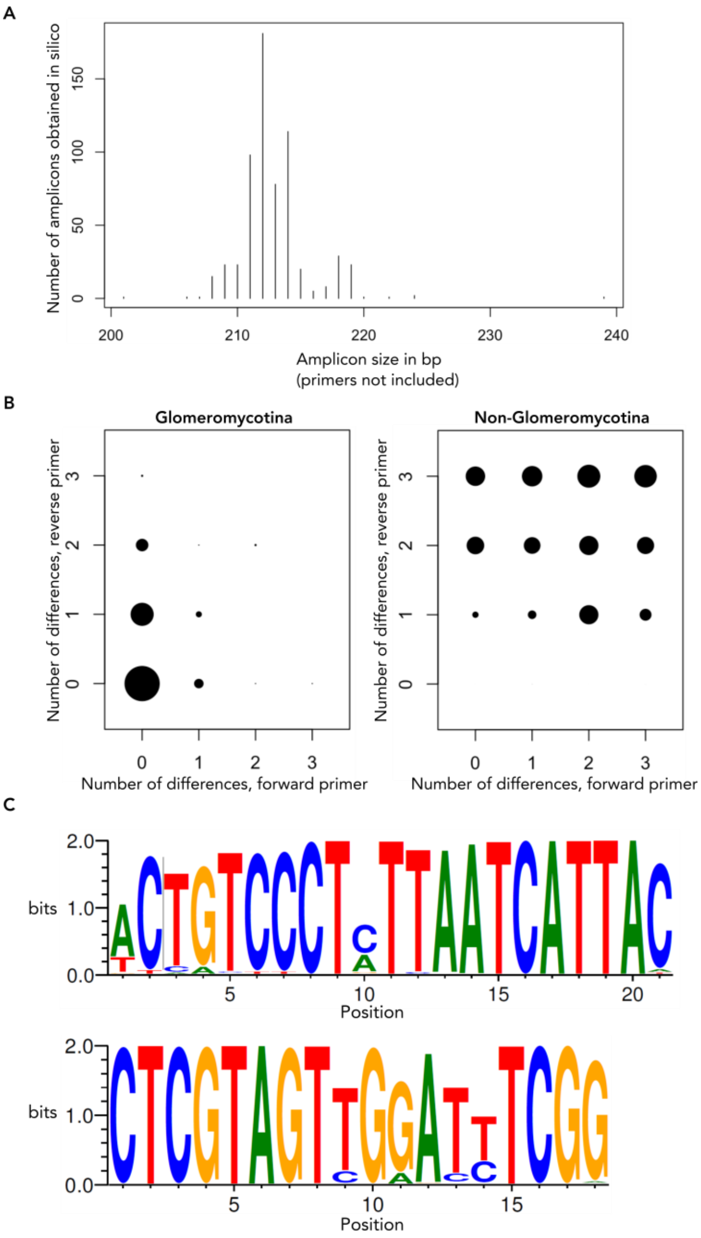

Fig. S2: Conservation and specificity of the Glom01 primer set for AM fungi. (A) Size distribution of the targeted amplicon. (B) Number of mismatches between Glom01 primers and target sequences, with circle size representing the number of taxa. (C) Sequence logo of Glom01 primers, where overall stack height represents sequence conservation at each position, individual symbol height indicates the relative frequency of each nucleotide or amino acid, and stack width corresponds to the fraction of valid symbols at that position. The logo was generated using WebLogo 3.3. Data provided courtesy of Argaly (Sainte-Hélène-du-Lac, France; https://www.argaly.com/).

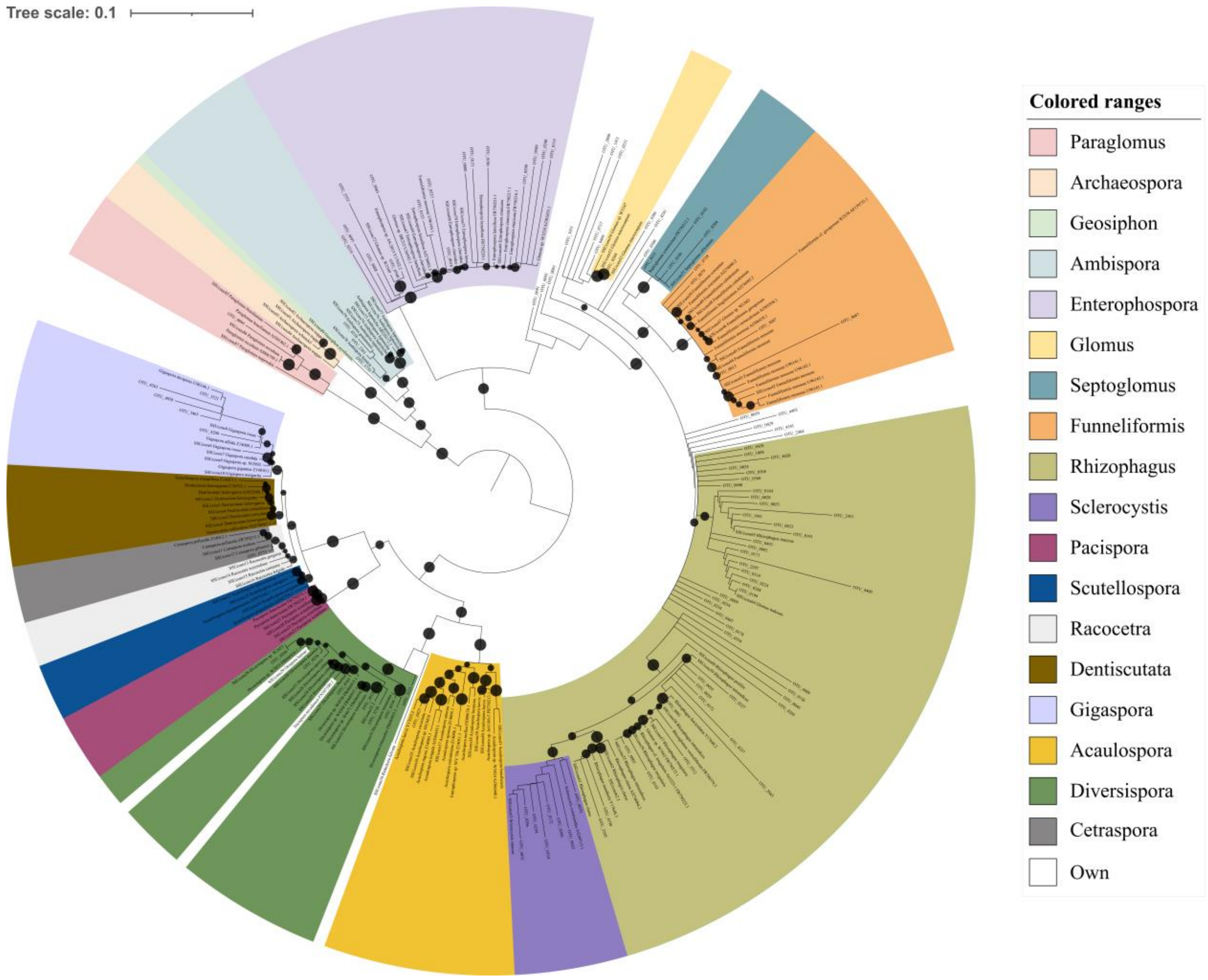

Fig. S3: Phylogenetic tree calculated using RAxML and evolutionary placement of OTU core sequences. Each OTU is classified taxonomically at the genus and/or family level. Black dots on the nodes indicate bootstrap values greater than 70, with the size of the dots representing the magnitude of these values (ranging from 70% to 100%). The scale bar denotes the branch length, corresponding to the expected number of substitutions per site. AM fungi with unclear phylogenetic position (for our OTU sequences) or taxonomy (for reference sequences) are labelled as “Own”.

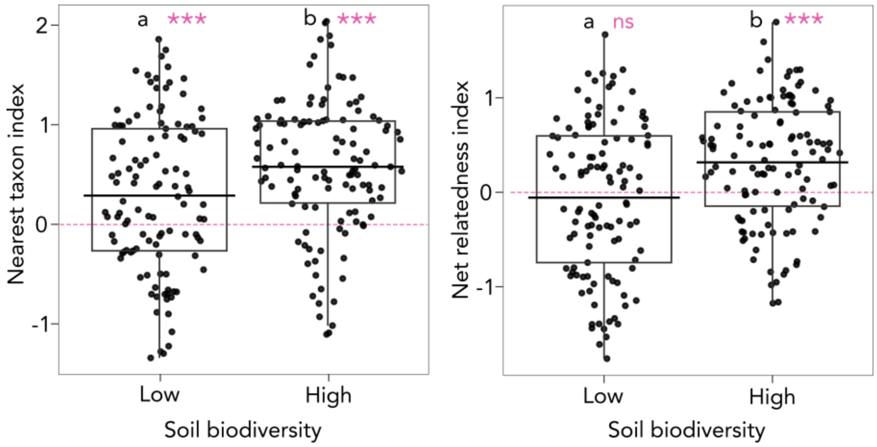

Fig. S4: Boxplots showing the effect of the soil biodiversity treatment on the nearest taxon index and net relatedness index for root-associated AM fungi (based on 18S metabarcoding profiles). The nearest taxon index represents the standardized mean phylogenetic distance to the nearest taxon, while the net relatedness index represents the standardized mean pairwise phylogenetic distance within each community. Both indices quantify the degree of phylogenetic relatedness among taxa within each plot. Significant differences between soil diversity treatments (Tukey's HSD) are indicated with letters, differences from zero (Student's t-Test) are indicated with asterisks (*** $p < 0.001$; ns >0.05).

Table S1: List of plant species used in the study and the experiments in which they were included. The experiment labelled "1" refers to the study examining the effects of plant and soil biodiversity under drought conditions, while "2" refers to the experiment investigating the impact of plant ecological strategy under ambient watering conditions, including plant monocultures and high soil biodiversity. Abbreviation: PFG – Plant Functional Group.

**Plant species**	**PFG**	**Plant family**	**Experiment**
***Leucanthemum vulgare* LAM.**	Forb	Asteraceae	1,2
***Centaurea jacea* L.**	Forb	Asteraceae	2
***Taraxacum officinale* (L.) WEBER**	Forb	Asteraceae	1,2
***Plantago lanceolata* L.**	Forb	Plantaginaceae	2
***Plantago media* L.**	Forb	Plantaginaceae	1,2
***Rumex acetosa* L.**	Forb/NonAM	Polygonaceae	2
***Brachypodium pinnatum* (L.) P.BEAUV.**	Grass	Poaceae	2
***Trisetum flavescens* (L.) P.BEAUV.**	Grass	Poaceae	1,2
***Dactylis glomerata* L.**	Grass	Poaceae	2
***Lolium perenne* L.**	Grass	Poaceae	1,2
***Avenula pubescens (*HUDS.) DUMORT.**	Grass	Poaceae	1,2
***Poa pratensis* L.**	Grass	Poaceae	2
***Lathyrus pratensis* L.**	Legume	Fabaceae	2
***Vicia cracca* L.**	Legume	Fabaceae	1,2
***Medicago sativa* L.**	Legume	Fabaceae	2
***Trifolium pratense* L.**	Legume	Fabaceae	1,2

Table S2: List of plant traits measured after three months and their ecological relevance.

Trait category	Plant trait	Definition	Abbr.	Ecological significance	Strategy	References
**Leaf morphological**	Specific leaf area[a]	Leaf area/leaf dry mass [m^2 kg^{-1}]	SLA	Higher SLA reflects greater metabolic activity and photosynthetic capacity, supporting an acquisitive strategy with quick resource returns but shorter lifespan.	Photosynthetic capacity, leaf construction and defence	(Pérez-Harguindeguy *et al.*, 2016; https://doi.org/10.1071/BT12225_CO)
	Leaf dry-matter content	Leaf dry mass/leaf fresh mass [mg g^{-1}]	LDMC	Opposite to SLA, higher LDMC indicates greater investment per unit of leaf volume and a slower growth strategy.	Leaf construction and defence	
**Leaf chemical**	Leaf nitrogen concentration[a]	Leaf Nitrogen content/dry mass [mg g $^{-1}$]	LNC	High LNC is related to greater photosynthetic capacity.	Photosynthetic capacity	(Pérez-Harguindeguy *et al.*, 2016)
**Plant architectural**	Leaf area index	Leaf area per ground area [unitless]	LAI	Increased LAI represents a greater investment in photosynthetic capacity and is linked to a plant's competitive ability.	Photosynthetic capacity	Calculated with SLA and leaf mass.
	Leaf mass fraction	Leaf dry mass/total plant dry mass [unitless]	LMF	Standing biomass distribution reflects plant physiology and growth rate, offering insights into responses to environmental changes. Increased LMF represents like LAI greater investment in photosynthetic capacity.	Resource allocation strategy, photosynthetic capacity	(Pérez-Harguindeguy *et al.*, 2016)
	Plant dry weight	Standing dry biomass [g]	-	Higher plant dry weight reflects strong performance and photosynthetic capacity, indicating competitive ability.	Photosynthetic capacity, plant performance	
**Root morphological**	Root tissue density[b]	Root dry mass per fresh root volume [g cm^{-3}]	RTD	Higher RTD signifies a greater investment per unit of root volume, aligning with a conservative strategy that prioritizes durability and slow, long-term returns.	Root construction and defence	(Freschet *et al.*, 2021; https://doi.org/10.1111/nph.17572)
	Root nitrogen concentration[b]	Root Nitrogen content/dry mass [mg g^{-1}]	RNC	Higher RNC reflects greater metabolic activity and nutrient uptake, supporting an acquisitive strategy with quick resource returns but shorter lifespan.	Metabolic activity, resource capture	
	Specific root length[b]	Root length/root dry mass [m g^{-1}]	SRL	Higher SRL indicates more efficient soil exploration per unit of root mass investment and reflects a "do-it-yourself" strategy for resource uptake.	Resource capture	
	Mean root diameter[b]	Root average diameter [mm]	MRD	MRD reflects root cortex and stele thickness, with thicker roots linked to an "outsourcing" strategy that depends more on mycorrhizal partners for resource uptake.	Resource capture, transport and root construction	
	Root hair length	Length of fully grown root hairs [mm]	RHL	Longer RHL helps plants access less mobile soil resources, offering an alternative nutrient uptake strategy independent of mycorrhizal fungi.	Resource capture	
	Root hair density	Number of root hairs/unit root length [unitless]	RHD	Like RHL, increased RHD indicates greater nutrient uptake capacity. Excessive density can lead to root hair competition, lowering efficiency.	Resource capture	
**Root architectural**	Mean rooting depth	Depth at which half of root mass occurs above [cm]	-	Mean rooting depth relates to a plant's below-ground zone of influence, limiting resource uptake capacities.	Resource capture, soil exploitation abilities	(Freschet *et al.*, 2021)
	Fine root mass fraction	Fine root dry mass per total plant standing dry biomass [unitless]	fRMF	Higher fRMF is linked to increased soil resource uptake capacities.	Resource capture and allocation	
	Shoot-to-root ratio	Aboveground plant dry mass/total plant dry mass [unitless]	SR-ratio	Higher SR-ratios indicate proportional increased aboveground biomass allocation, which can be influenced by factors such as mycorrhizal collaboration and nutrient availability.	Resource capture and allocation	
	Root length density	Root length per soil volume [m]	RLD	Higher RLD is linked to increased soil resource uptake capacities and soil exploration.	Resource capture, soil exploitation abilities	Calculated with SRL and total root dry mass.
**Mycorrhizal**	Mycorrhizal colonisation intensity	% of root length colonised by AM fungi [%]	MCI	Related to the degree of plant reliance on mycorrhizae.	Resource uptake, plant reliance on mycorrhiza	Line intersection method (Brundrett *et al.*, 1996; http://dx.doi.org/10.13140/2.1.4880.5444); 100 intersections/slide
	Arbuscule to Vesicle Ratio	N°arbuscules/ N°vesicles [unitless]	-	Related to the intensity of exchange between partners	Resource exchange with mycorrhizae	

a: Leaf traits used in a principal component analysis (PCA) to define the leaf economic spectrum (leaf PC1)

b: Root traits used in a PCA to define the root economic space, composed of the root collaboration gradient (RCG1; root PC1, defined by SRL and MRD) and the root conservation gradient (RCG2; root PC2, defined by RNC and RTD)

Table S3: Specificity and taxonomic resolution for targeting AM fungi using two primer pairs: the Glom01 primer set (used in this study) and the commonly used NS31-AML2. Data provided courtesy of Argaly (Sainte-Hélène-du-Lac, France; https://www.argaly.com/).

**Primer set**	**Glom01**		**NS31-AML2**	
**Species amplified**	625		276	
**Species non-amplified**	43833		52786	
	**identified**	**amplified**	**identified**	**amplified**
**Class**	0	1	0	1
**Order**	2	4	2	4
**Family**	6	11	6	11
**Genus**	10	24	15	23
**Species**	338	625	196	276

Table S4: Correlation between the final selected traits used to model the richness of AM fungal communities and its variability.

	**RCG1**	**RHL**	**LDMC**	**SR-ratio**
**RCG1**	1	-0.24	-0.03	0.16
**RHL**		1	0.21	-0.28
**LDMC**			1	-0.33

Table S5: Analysis of deviance table for AM fungal OTU richness (OR; Type 2), phylogenetic richness calculated based on RAxML-ng phylogeny (PR; Type 3) and AM fungal phylogenetic richness calculated based on evolutionary placement (EPA algorithm) on reference phylogeny (PR_s; Type 3; sensitivity analysis).

**Effector**	**Response**	**Chi-square**	**DF**	***P* value**	**Sig.**
**Drought**	OR	22.54	1	<0.001	***
**Soil biodiversity**	OR	21.17	1	<0.001	***
**Plant diversity**	OR	6.49	1	0.011	*
**Soil biodiversity**	PR	14.63	1	<0.001	***
**Plant diversity**	PR	2.32	1	0.127	
**Drought**	PR	29.44	1	<0.001	***
**Soil biodiv. × Drought**	PR	4.96	1	0.026	*
**Plant div. × Drought**	PR	4.13	1	0.042	*
**Soil biodiversity**	PR_s	15.71	1	<0.001	***
**Plant diversity**	PR_s	4.65	1	0.031	*
**Drought**	PR_s	31.06	1	<0.001	***
**Soil biodiv. × Drought**	PR_s	3.98	1	0.046	*
**Plant div. × Drought**	PR_s	3.59	1	0.058	.

Table S6: Results of zero-inflated negative binomial GLM modelling including plant functional group (PFG), plant functional traits, and time (T1: four months, T2: 16 months), against AM fungal OTU Richness (OR), phylogenetic species richness calculated based on RAxML-ng phylogeny (PR) and AM fungal phylogenetic species richness calculated based on evolutionary placement (EPA algorithm) on a reference phylogeny (PR_s; sensitivity analysis).

Effector	Resp.	Estimate	Std. Error	z-value	*P* value	Sig.
**Intercept**	OR	1.34	**0.23**	**5.81**	<0.001	***
**PFG Forb**	OR	1.26	0.25	5.10	<0.001	***
**PFG Legume**	OR	0.74	0.27	2.76	0.006	**
**RHL**	OR	-5.96	1.43	-4.18	<0.001	***
**Time (15 months)**	OR	1.65	0.25	6.71	<0.001	***
**PFG Forb × Time**	OR	-1.19	0.27	-4.34	<0.001	***
**PFG Legume × Time**	OR	-1.14	0.30	-3.77	<0.001	***
**RHL × Time**	OR	5.38	1.50	3.60	<0.001	***
**Intercept**	PR	-0.07	0.26	-0.25	0.802	
**PFG Forb**	PR	1.18	0.27	4.37	<0.001	***
**PFG Legume**	PR	0.60	0.29	2.08	0.037	*
**RHL**	PR	-7.62	1.72	-4.44	<0.001	***
**Time (15 months)**	PR	1.55	0.28	5.47	<0.001	***
**PFG Forb × Time**	PR	-0.93	0.30	-3.05	0.002	**
**PFG Legume × Time**	PR	-1.02	0.34	-2.97	0.003	**
**RHL × Time**	PR	7.25	1.81	4.01	<0.001	***
**Intercept**	PR_s	0.23	0.27	0.85	0.398	
**PFG Forb**	PR_s	1.21	0.27	4.41	<0.001	***
**PFG Legume**	PR_s	0.71	0.29	2.45	0.014	*
**RHL**	PR_s	-0.80	0.20	-4.05	<0.001	***
**Time (15 months)**	PR_s	1.64	0.28	5.78	<0.001	***
**PFG Forb × Time**	PR_s	-1.01	0.30	-3.33	<0.001	***
**PFG Legume × Time**	PR_s	-1.17	0.33	-3.50	<0.001	***
**RHL × Time**	PR_s	0.72	0.21	3.45	<0.001	***

Table S7: Results of zero-inflated negative binomial GLM modelling for plant functional traits against AM fungal OTU Richness (OR), phylogenetic species richness calculated based on RAxML-ng phylogeny (PR), and AM fungal phylogenetic richness calculated based on evolutionary placement (EPA algorithm) on reference phylogeny (PR_s; sensitivity analysis). RCG1 describes the root collaboration gradient.

Effector	Resp.	Estimate	Std. Error	z-value	*P*-value	Sig.
**Intercept**	OR	1.89	0.12	15.76	<0.001	
**RHL**	OR	-0.72	0.16	-4.64	<0.001	***
**LDMC**	OR	-0.40	0.09	-4.30	<0.001	***
**RCG1**	OR	0.38	0.11	3.56	<0.001	***
**Time (15 months)**	OR	1.01	0.13	7.71	<0.001	***
**SR-ratio**	OR	-0.09	0.04	-2.15	0.032	*
**RHL × Time**	OR	0.64	0.17	3.83	<0.001	***
**LDMC × Time**	OR	0.25	0.11	2.33	0.020	*
**RCG1 × Time**	OR	-0.56	0.12	-4.52	<0.001	***
**Intercept**	PR	0.54	0.14	3.82	<0.001	***
**RHL**	PR	-0.77	0.18	-4.19	<0.001	***
**LDMC**	PR	-0.45	0.10	-4.30	<0.001	***
**RCG1**	PR	0.29	0.13	2.24	0.025	*
**Time (15 months)**	PR	0.91	0.16	5.90	<0.001	***
**SR-ratio**	PR	-0.13	0.05	-2.63	0.009	**
**RHL × Time**	PR	0.65	0.20	3.28	0.001	**
**LDMC × Time**	PR	0.22	0.12	1.80	0.073	.
**RCG1 × Time**	PR	-0.44	0.15	-2.94	0.003	**
**Intercept**	PR_s	0.82	0.15	5.55	<0.001	***
**RHL**	PR_s	-0.76	0.18	-4.16	<0.001	***
**LDMC**	PR_s	-0.44	0.11	-4.06	<0.001	***
**RCG1**	PR_s	0.34	0.13	2.53	0.011	*
**Time (15 months)**	PR_s	0.98	0.16	6.09	<0.001	***
**SR-ratio**	PR_s	-0.12	0.05	-2.47	0.014	*
**RHL × Time**	PR_s	0.65	0.20	3.33	<0.001	***
**LDMC × Time**	PR_s	0.23	0.12	1.82	0.069	.
**RCG1 × Time**	PR_s	-0.53	0.15	-3.55	<0.001	***

Table S8: Pagel's λ assessing the phylogenetic signal for all variables related to Experiment 2, which includes all 16 plant species.

	**Predictor**	**Pagel's λ**	***P*-value**
**Diversity indices**	Phylogenetic richness	0.23	0.399
	OTU richness	0.38	0.174
	Temporal variability (%)	0.39	0.131
**Plant traits**	RCG1	0.85	0.001
	RHL	0.00	1.000
	LDMC	0.78	0.004
	SR-ratio	0.00	1.000

Table S9: Phylogenetic linear mixed models testing the relationship between plant traits and AM fungal diversity while accounting for plant phylogenetic relatedness. Plant phylogenetic structure is incorporated as a random intercept variable. Models have been calculated only for Experiment 2, which includes all 16 plant species. Phylogenetic richness was log-transformed to approximate a normal distribution.

	**Predictor**	**Value**	**Std. Error**	**Z-Score**	***P* value**	**Sig.**
**Phylogenetic richness**	Intercept	0.62	0.13	4.94	<0.001	***
	RHL	-0.71	0.17	-4.24	<0.001	***
	RCG1	0.26	0.12	2.23	0.026	*
	Time	0.82	0.14	5.95	<0.001	***
	SR-ratio	-0.14	0.05	-2.68	0.007	**
	LDMC	-0.30	0.06	-5.21	<0.001	***
	RHL × Time	0.59	0.18	3.22	0.001	**
	RCG1 × Time	-0.43	0.14	-3.04	0.002	**
**Temporal variability (%)**	Intercept	0.55	0.51	1.09	0.275	
	RCG1	-0.32	0.11	-2.84	0.004	**
	RHL	0.84	0.41	2.04	0.041	*

Table S10: Results of the RDA model selection: The condition "PFG" includes model selection incorporating plant functional groups and traits, while the "PFT" condition utilizes only plant functional traits.

Predictor	Condition	R^2 adj.	R^2 predictor	Df	F	Pr(>F)	Sig.
**PFG**	PFG	0.04	0.05	2	3.02	0.002	**
**Time**	PFG	0.07	0.04	1	4.31	0.002	**
**RHL**	PFG	0.08	0.02	1	1.58	0.032	*
**All variables**	PFG	0.09					
**Time**	PFT	0.04	0.04	1	4.51	0.002	**
**RCG1**	PFT	0.05	0.02	1	2.59	0.002	**
**RCG2**	PFT	0.06	0.02	1	2.27	0.002	**
**RHL**	PFT	0.07	0.02	1	1.72	0.006	**
**LDMC**	PFT	0.08	0.02	1	1.63	0.01	**
**All variables**	PFT	0.10					

Table S11: Pairwise differences in dispersion between the three plant functional groups, assessed using Tukey's test at three months, 15 months, and across both time points.

Pair	Time	Difference	Lower CI	Upper CI	p adjusted
**herb-grass**	both	-0.72	-1.17	-0.28	<0.001
**legume-grass**	both	-1.06	-1.53	-0.59	<0.001
**legume-herb**	both	-0.34	-0.81	0.14	0.217
**herb-grass**	3 months	-1.05	-1.71	-0.40	0.001
**legume-grass**	3 months	-1.12	-1.79	-0.44	<0.001
**legume-herb**	3 months	-0.06	-0.70	0.57	0.968
**herb-grass**	15 months	-0.57	-1.10	-0.05	0.029
**legume-grass**	15 months	-0.63	-1.20	-0.06	0.027
**legume-herb**	15 months	-0.06	-0.65	0.54	0.972
